# Supplementary figures and images for: Control of 3′ splice site selection by the yeast splicing factor Fyv6
Source: eLife. 2024 Dec 17;13:RP100449. doi: 10.7554/eLife.100449 (PMC11651659; doi:10.7554/eLife.100449)

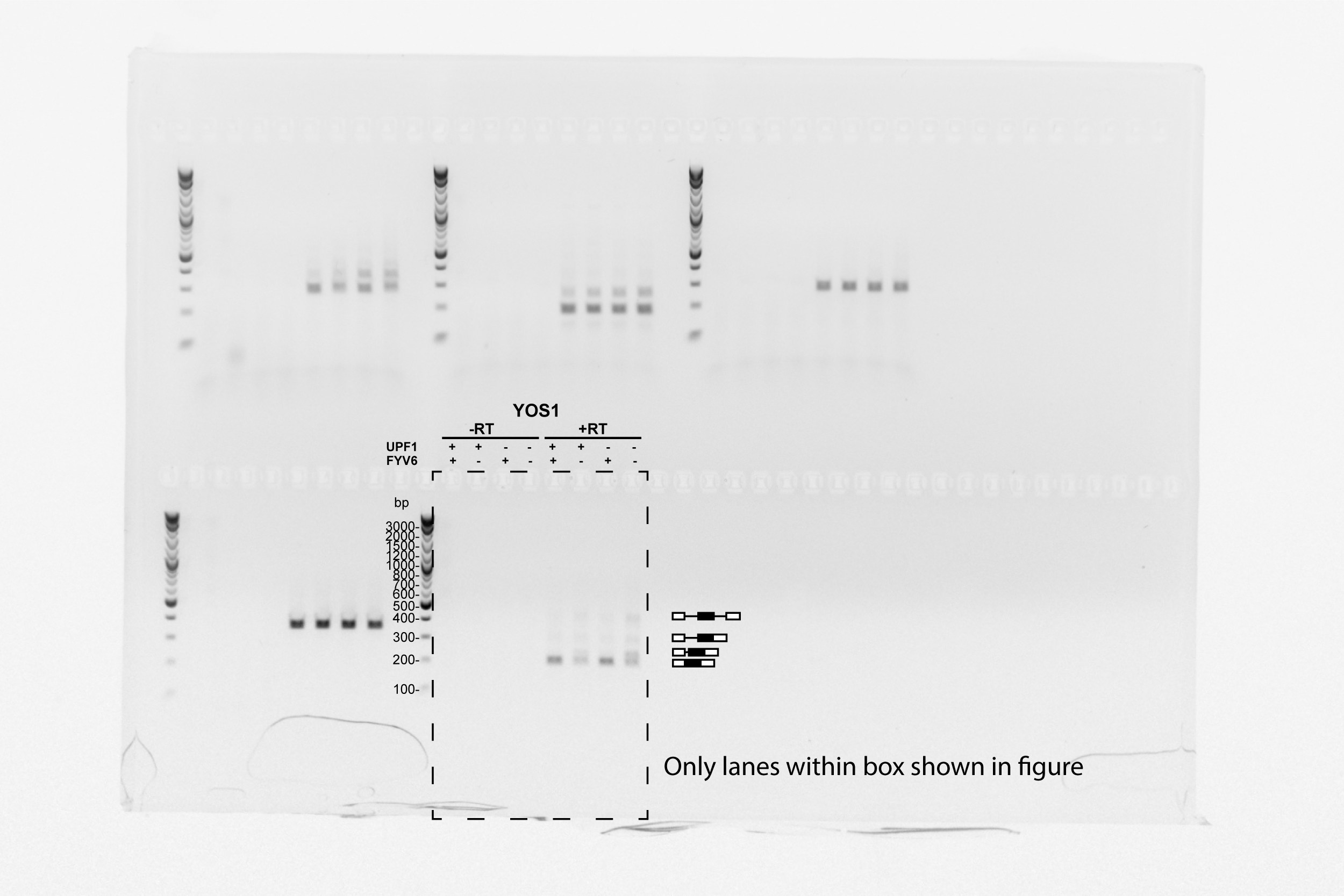

Supplement: Figure 1—figure supplement 1—source data 1. [file elife-100449-fig1-figsupp1-data1.zip › Figure 1-figure supplement 1-source data 1/Figure1-FigureSupplement1-labeled.tif]

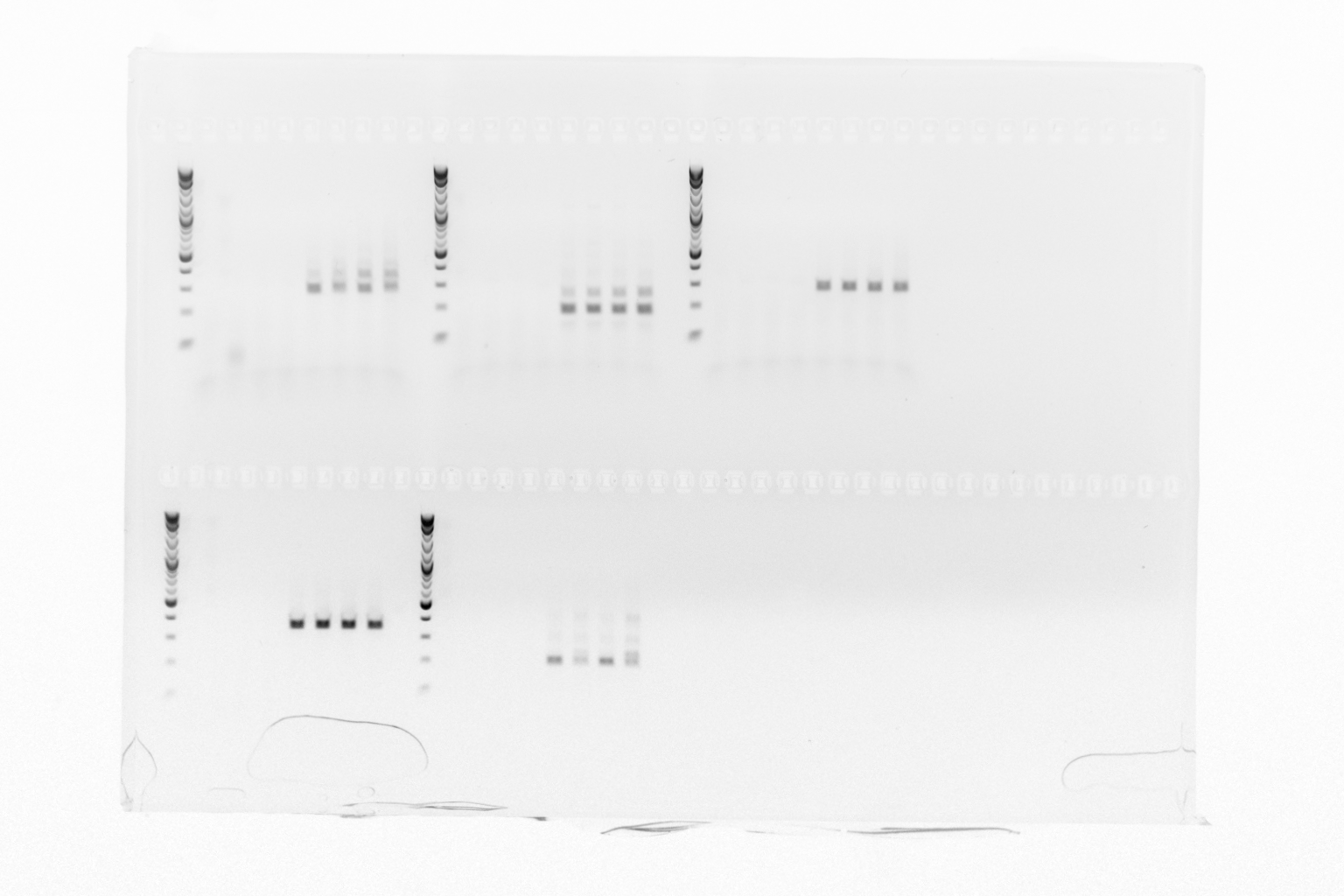

Supplement: Figure 1—figure supplement 1—source data 2. [file elife-100449-fig1-figsupp1-data2.zip › Figure 1-figure supplement 1-source data 2/Figure1-FigureSupplement1-raw.gel]

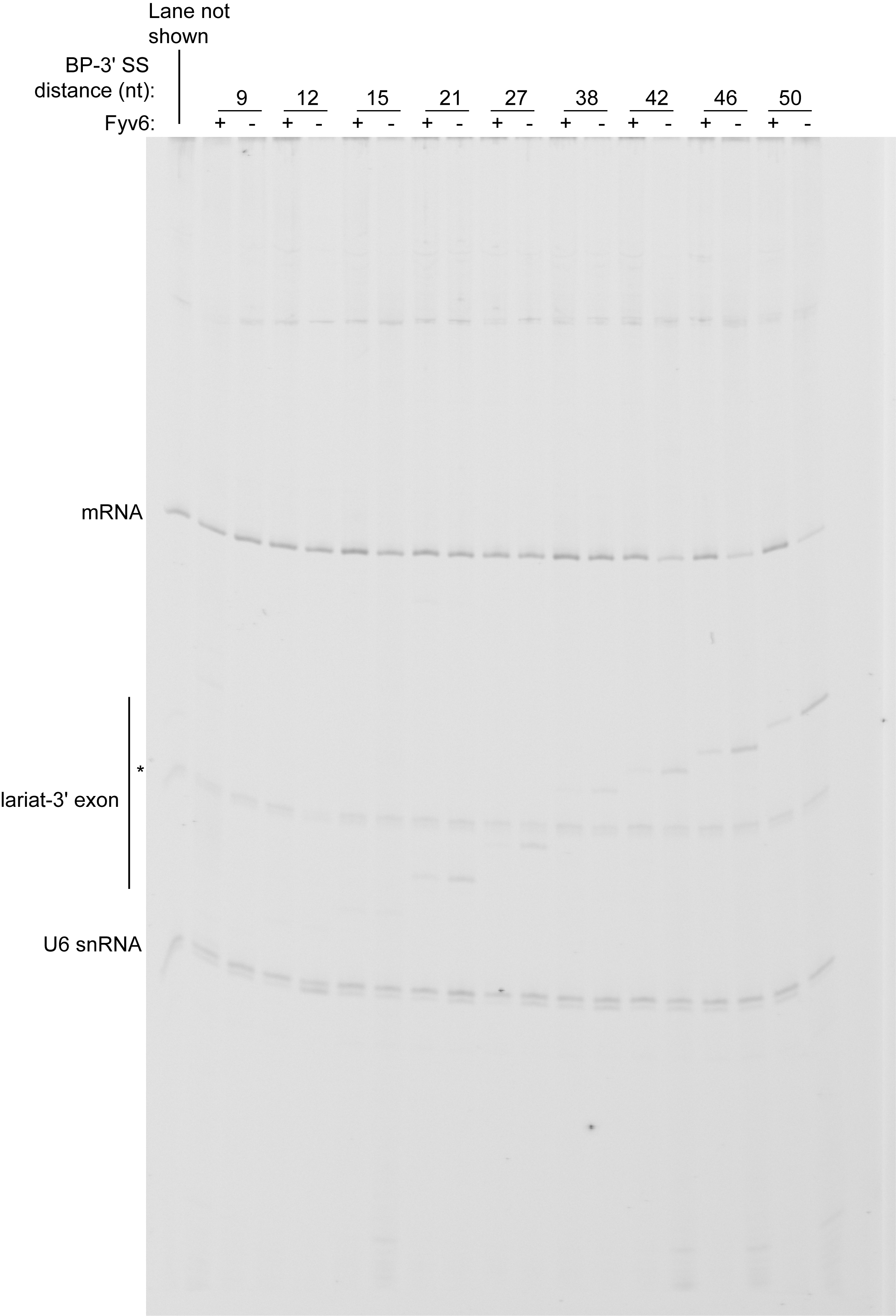

Supplement: Figure 2—source data 1. [file elife-100449-fig2-data1.zip › Figure 2-source data 1/Figure2e-labeled.tif]

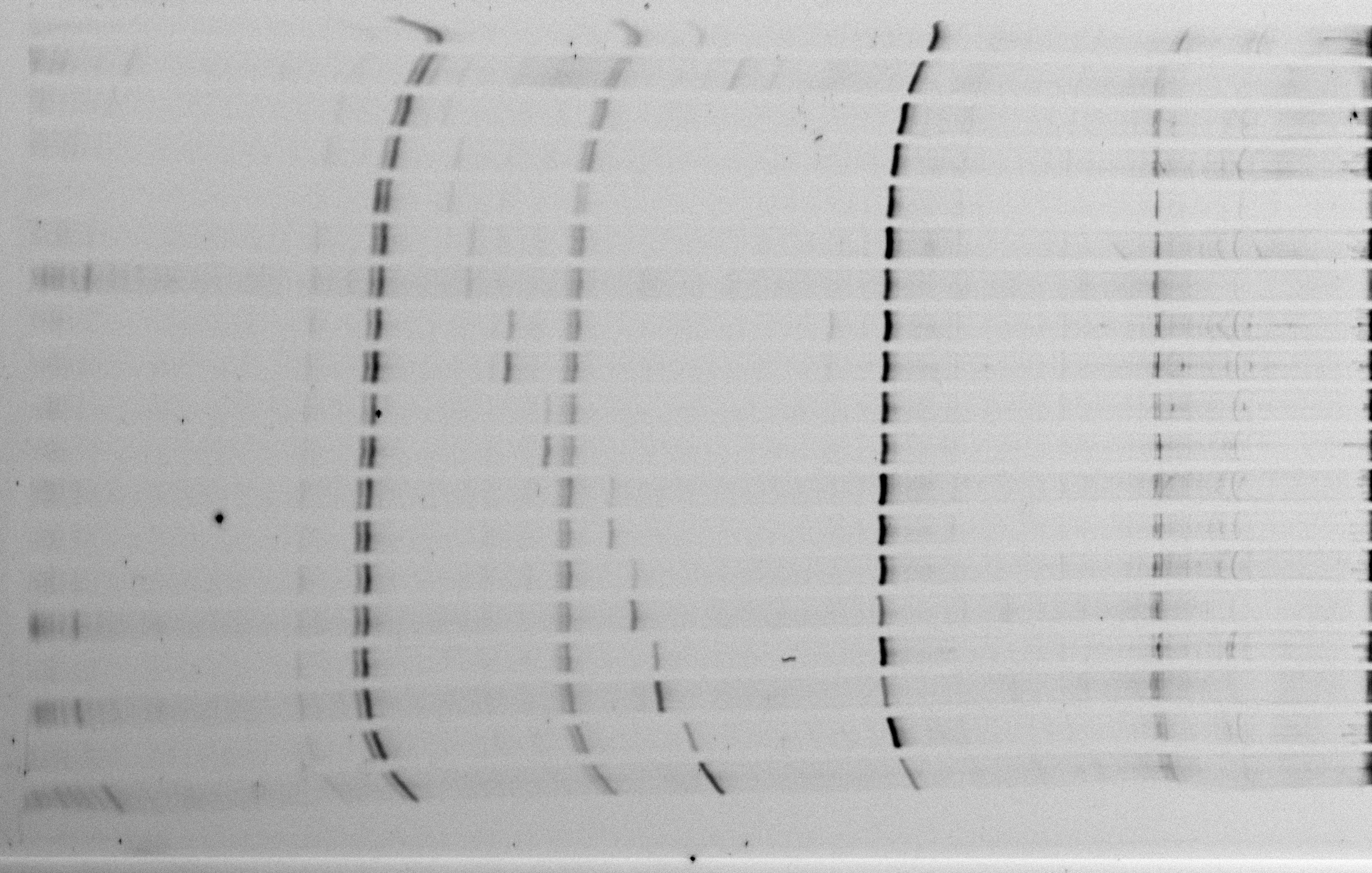

Supplement: Figure 2—source data 2. [file elife-100449-fig2-data2.zip › Figure 2-source data 2/Figure2e-raw.gel]

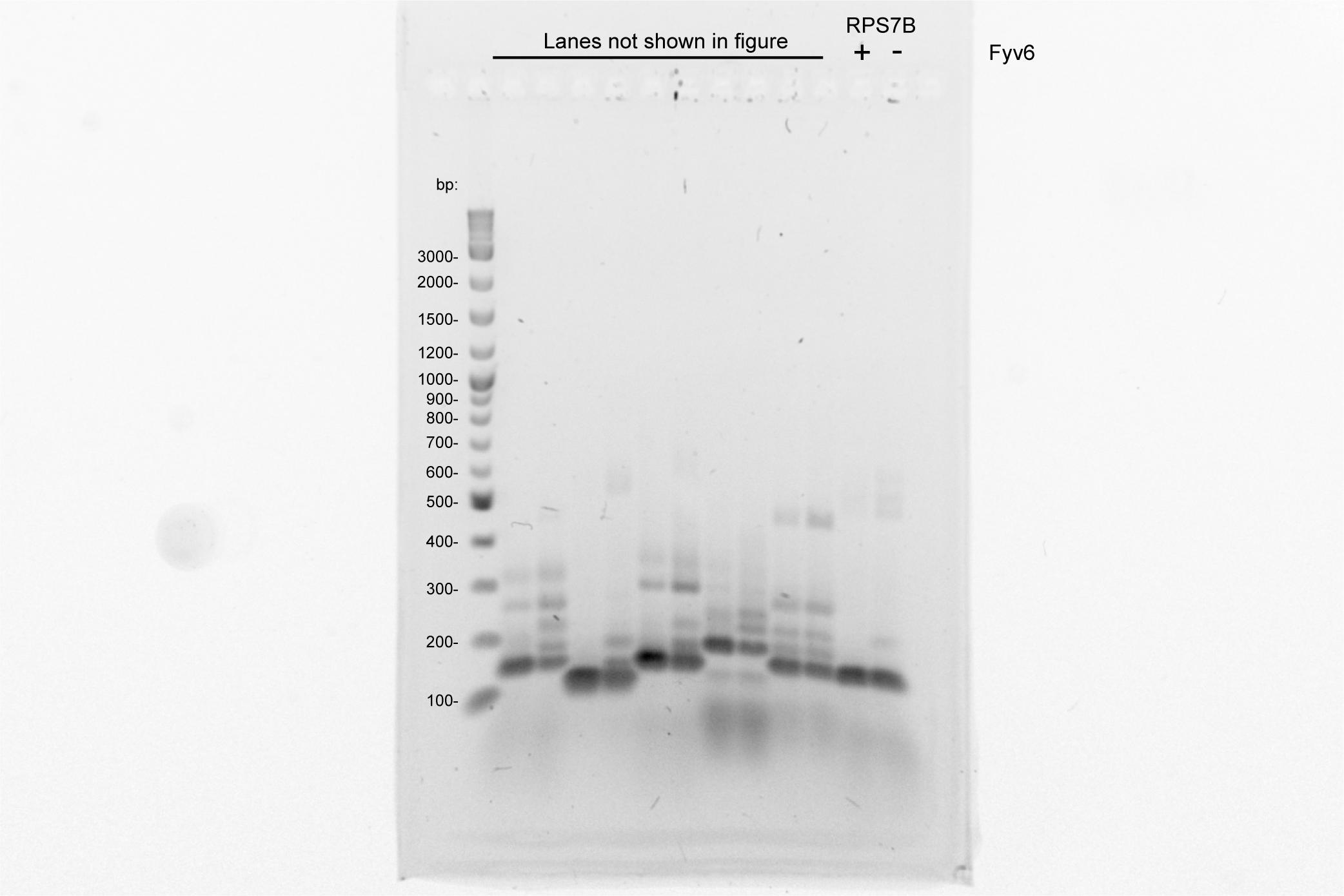

Supplement: Figure 2—figure supplement 1—source data 1. [file elife-100449-fig2-figsupp1-data1.zip › Figure 2-figure supplement 1-source data 1/Figure2-FigureSupplement1-labeled2.tif]

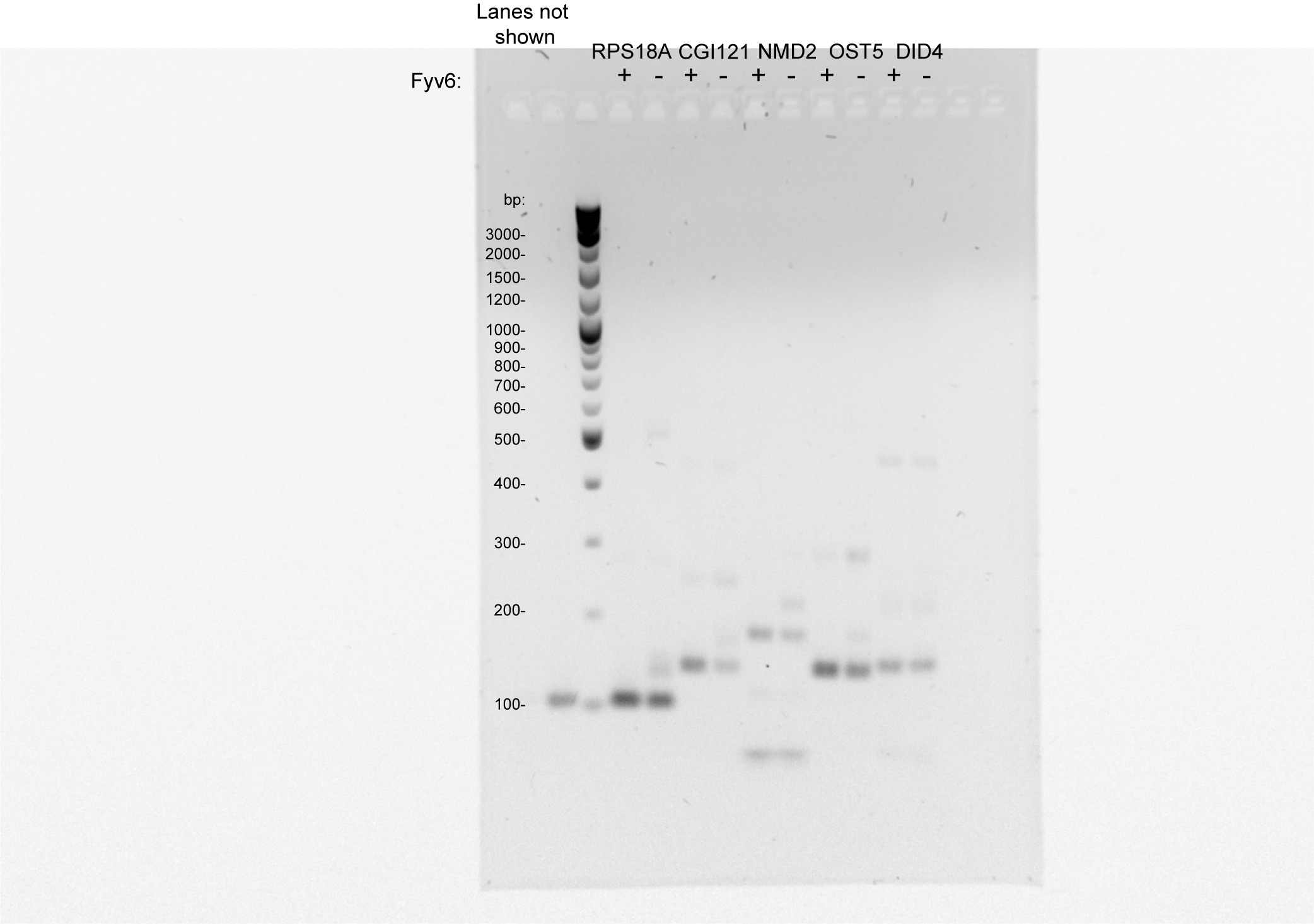

Supplement: Figure 2—figure supplement 1—source data 1. [file elife-100449-fig2-figsupp1-data1.zip › Figure 2-figure supplement 1-source data 1/Figure2-FigureSupplement1-labeled1.tif]

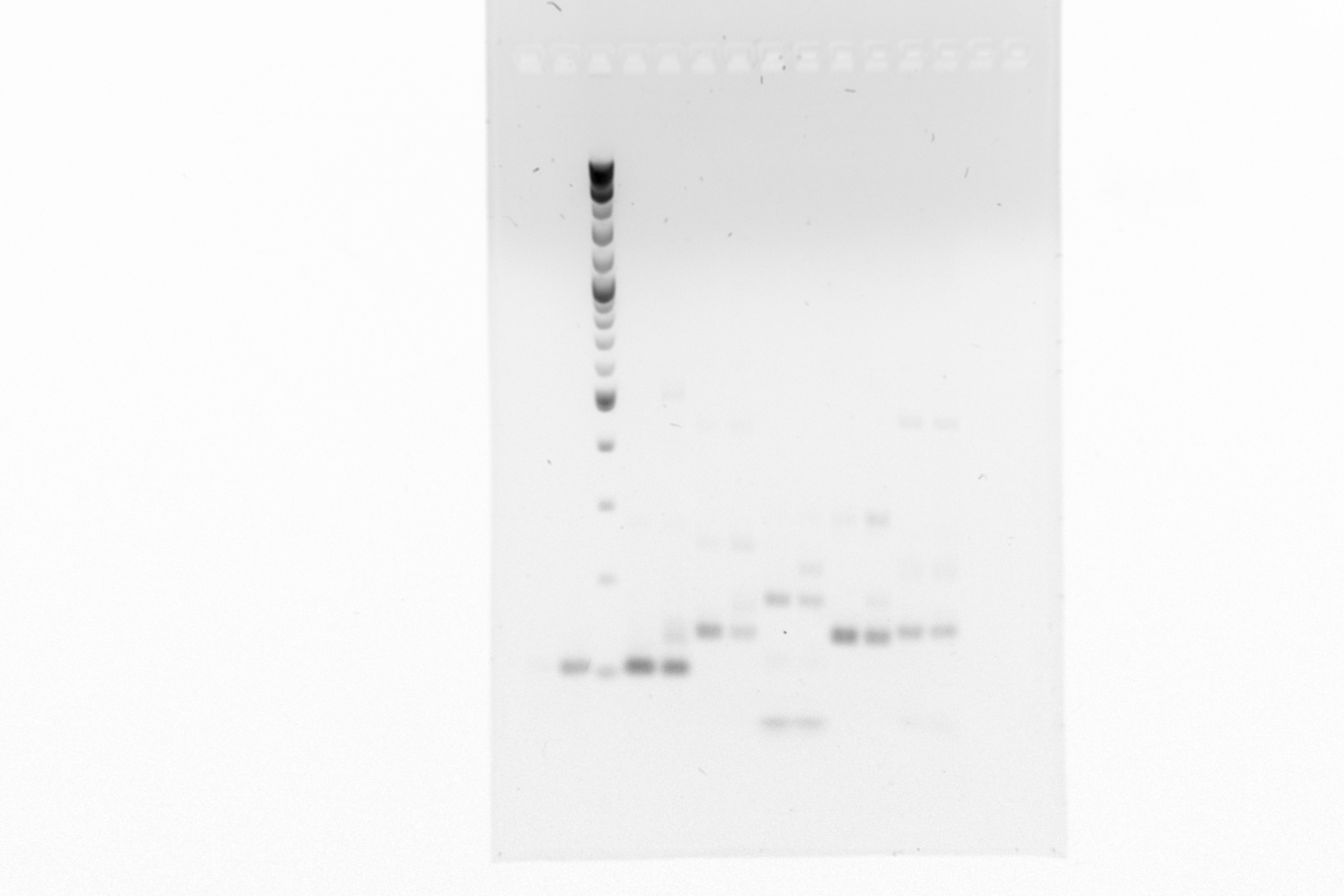

Supplement: Figure 2—figure supplement 1—source data 2. [file elife-100449-fig2-figsupp1-data2.zip › Figure 2-figure supplement 1-source data 2/Figure2-FigureSupplement1-raw1.gel]

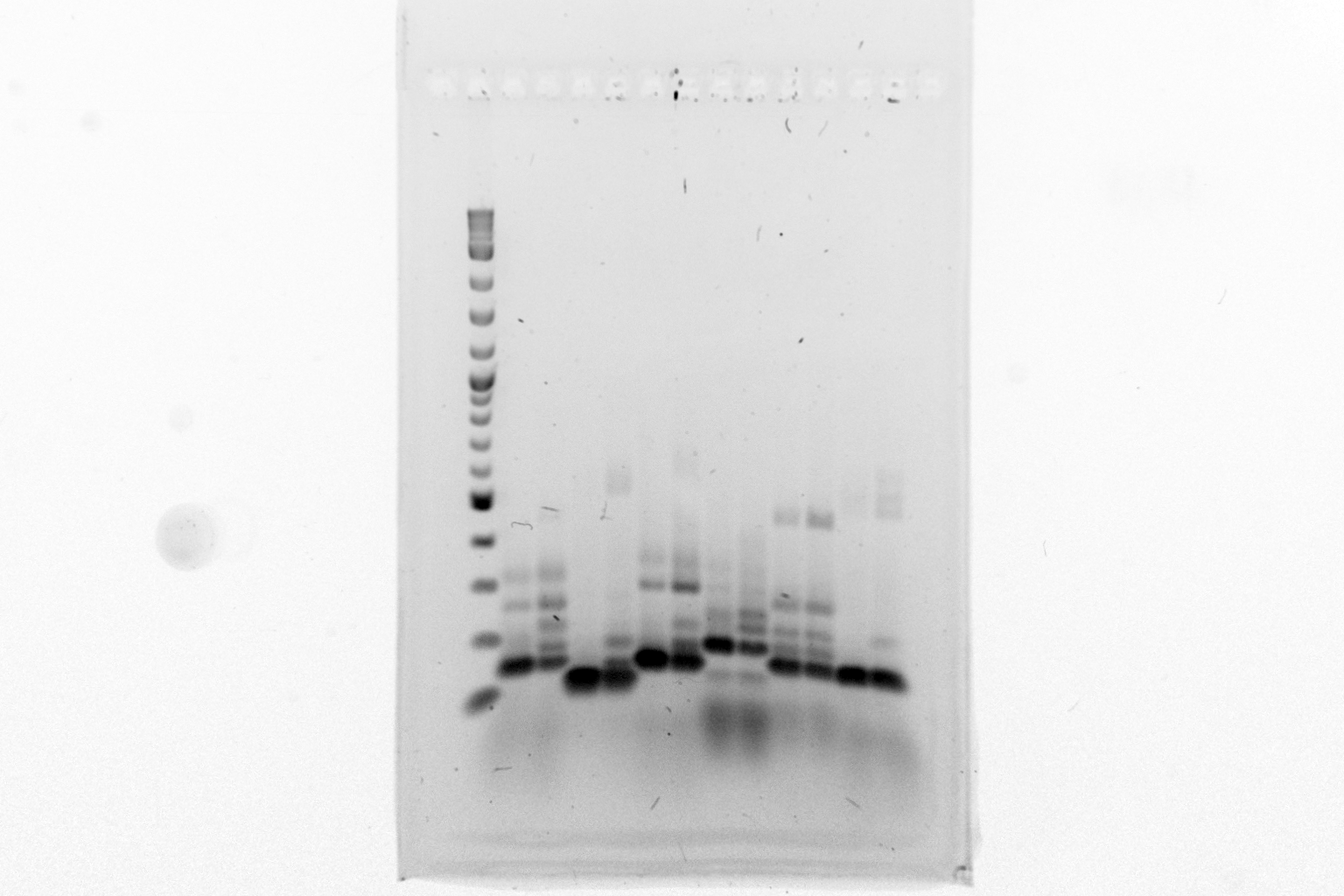

Supplement: Figure 2—figure supplement 1—source data 2. [file elife-100449-fig2-figsupp1-data2.zip › Figure 2-figure supplement 1-source data 2/Figure2-FigureSupplement1-raw2.gel]

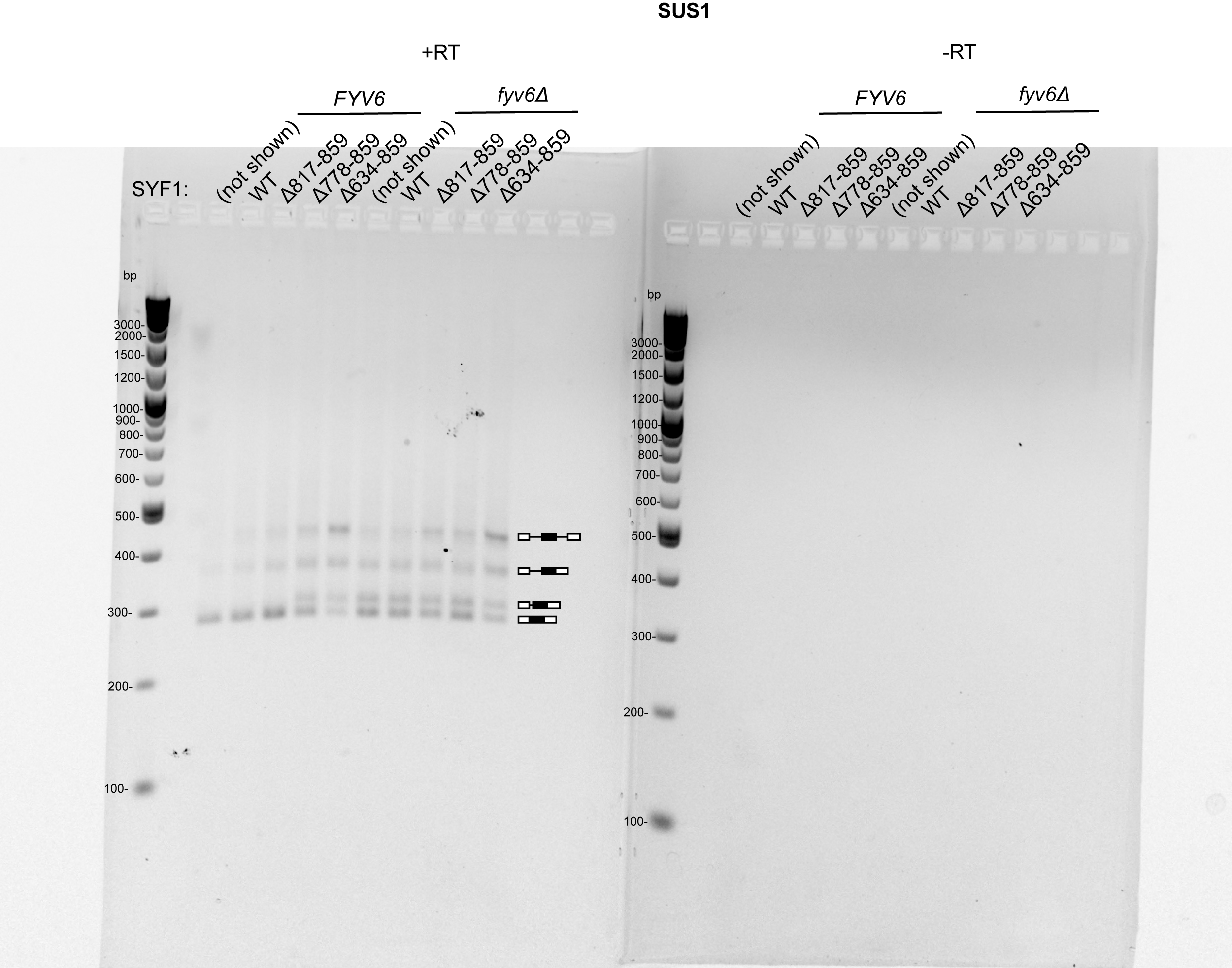

Supplement: Figure 5—source data 1. [file elife-100449-fig5-data1.zip › Figure 5-source data 1/Figure5F+H-labeled.tif]

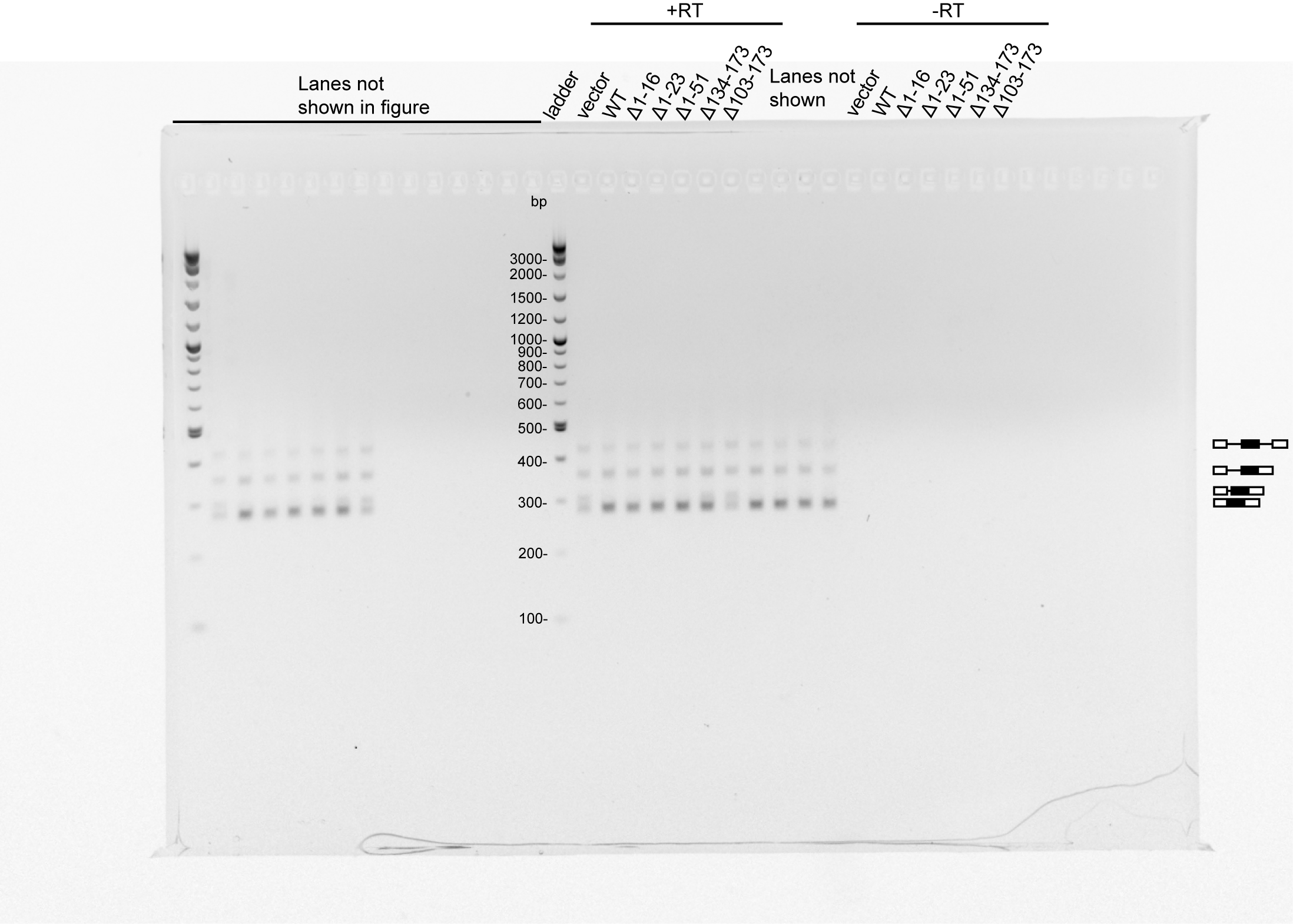

Supplement: Figure 5—source data 1. [file elife-100449-fig5-data1.zip › Figure 5-source data 1/Figure5c-labeled.tif]

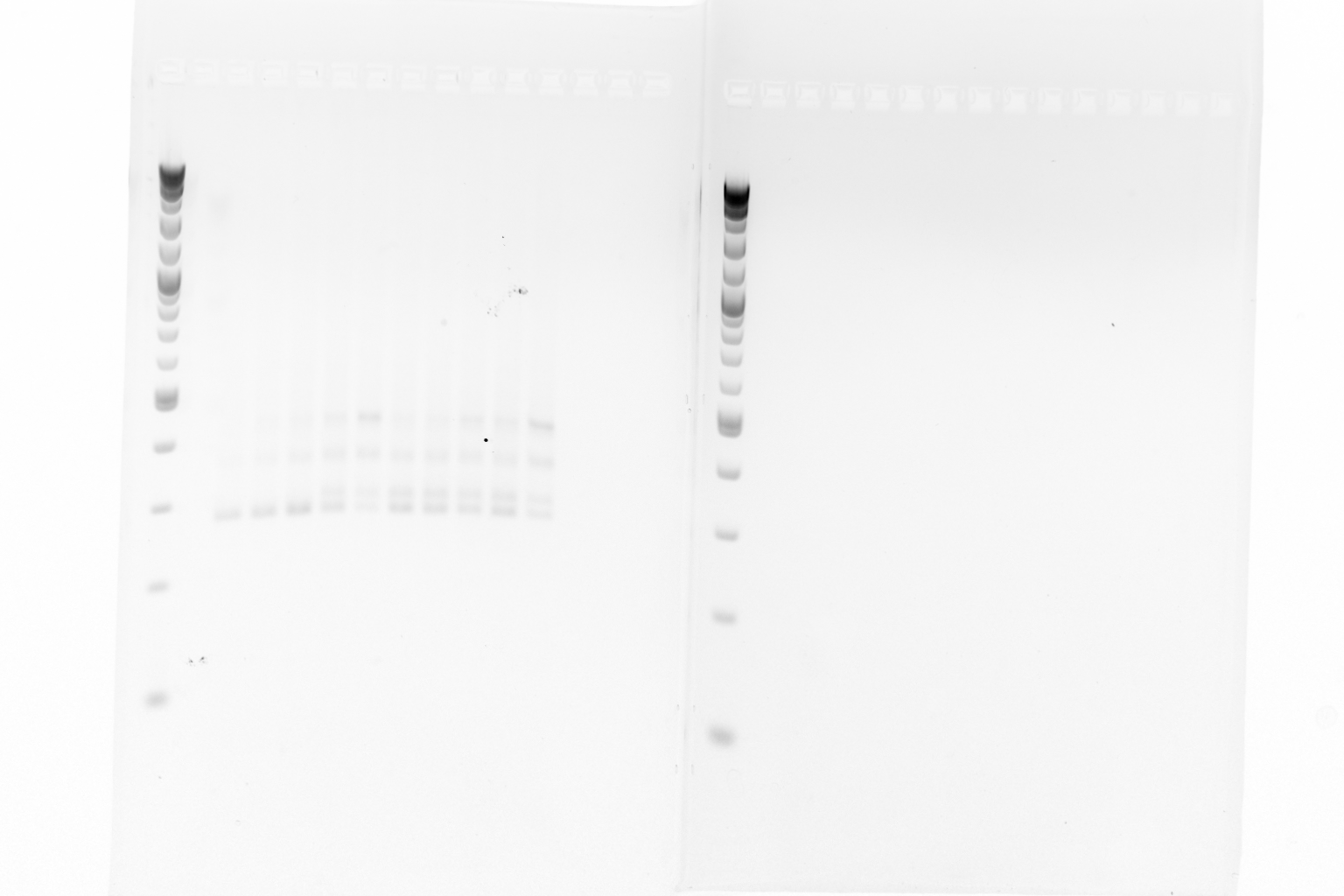

Supplement: Figure 5—source data 2. [file elife-100449-fig5-data2.zip › Figure 5-source data 2/Figure5F+H-raw.gel]

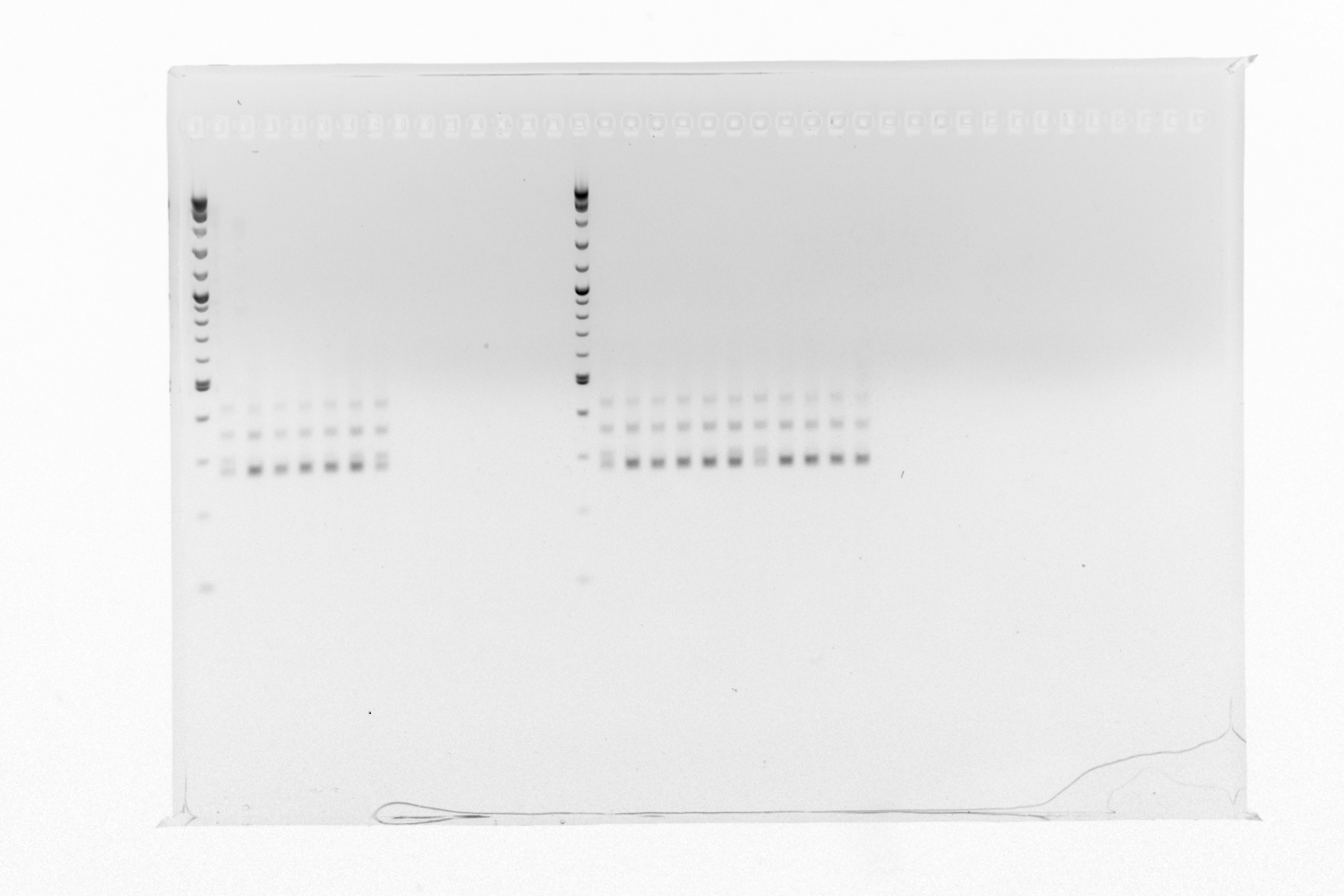

Supplement: Figure 5—source data 2. [file elife-100449-fig5-data2.zip › Figure 5-source data 2/Figure5c-raw.gel]

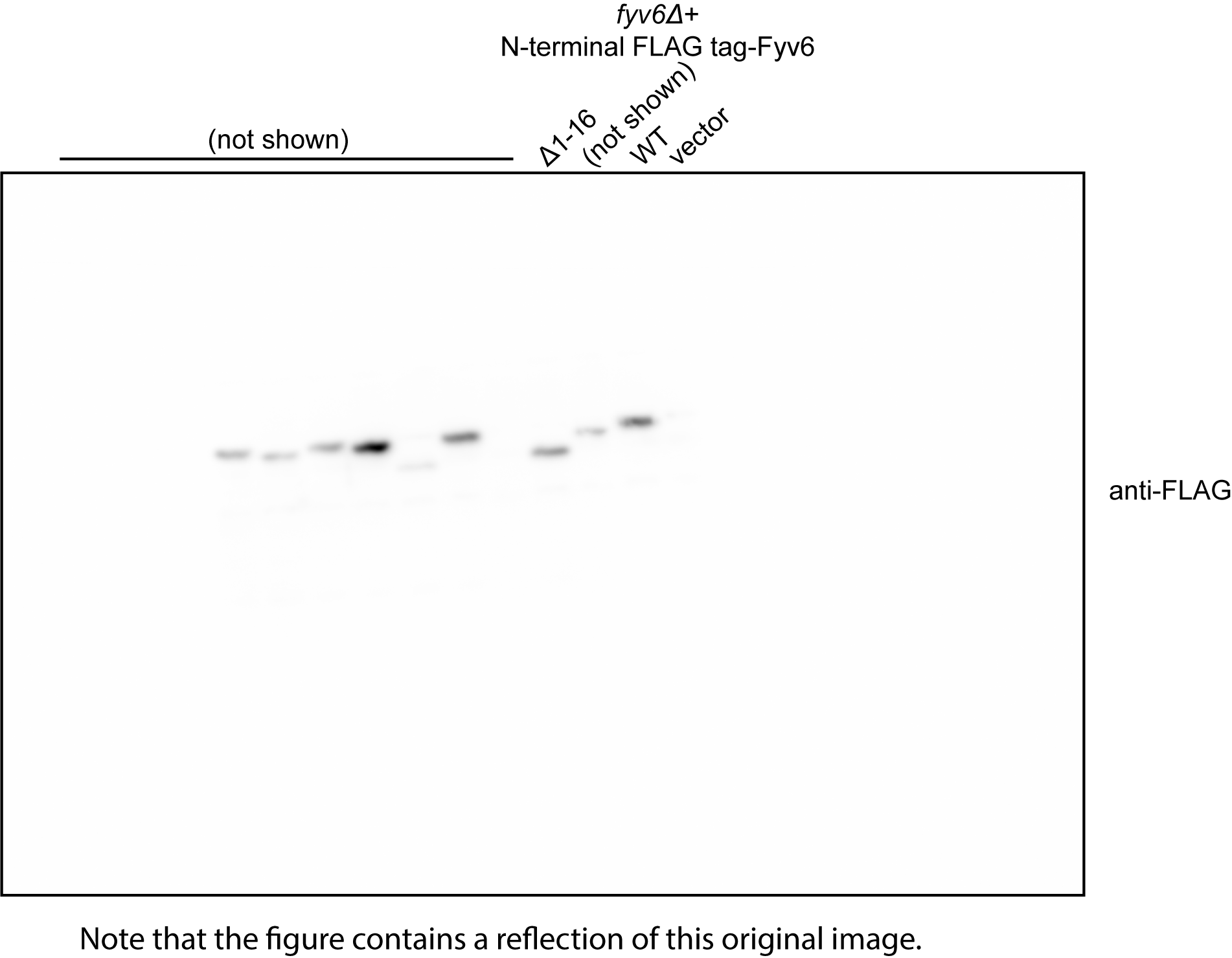

Supplement: Figure 5—figure supplement 1—source data 1. [file elife-100449-fig5-figsupp1-data1.zip › Figure 5-figure supplement 1-source data 1/Figure5-FigureSupplement1-FLAG-2-labeled.tif]

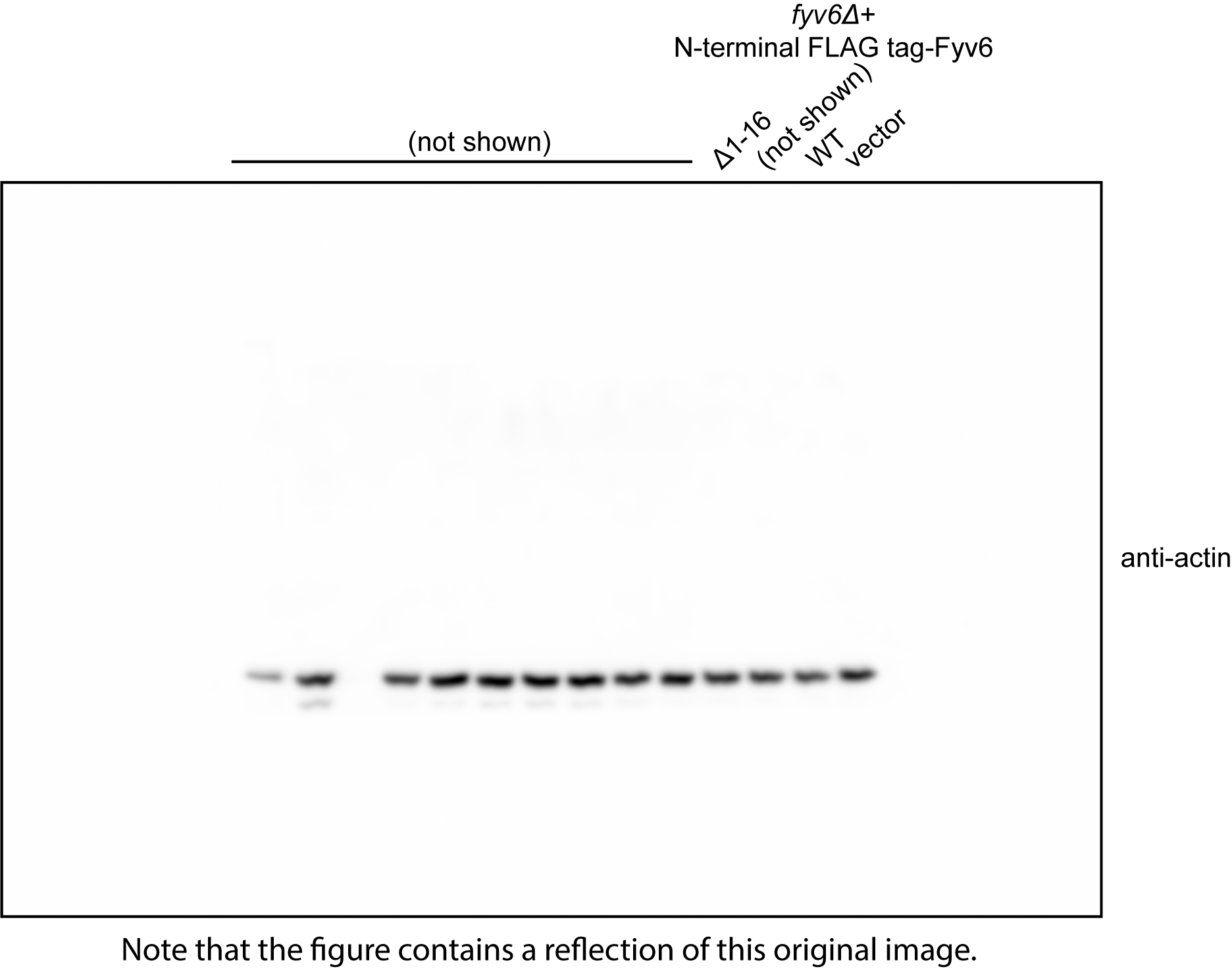

Supplement: Figure 5—figure supplement 1—source data 1. [file elife-100449-fig5-figsupp1-data1.zip › Figure 5-figure supplement 1-source data 1/Figure5-FigureSupplement1-Actin-2-labeled.tif]

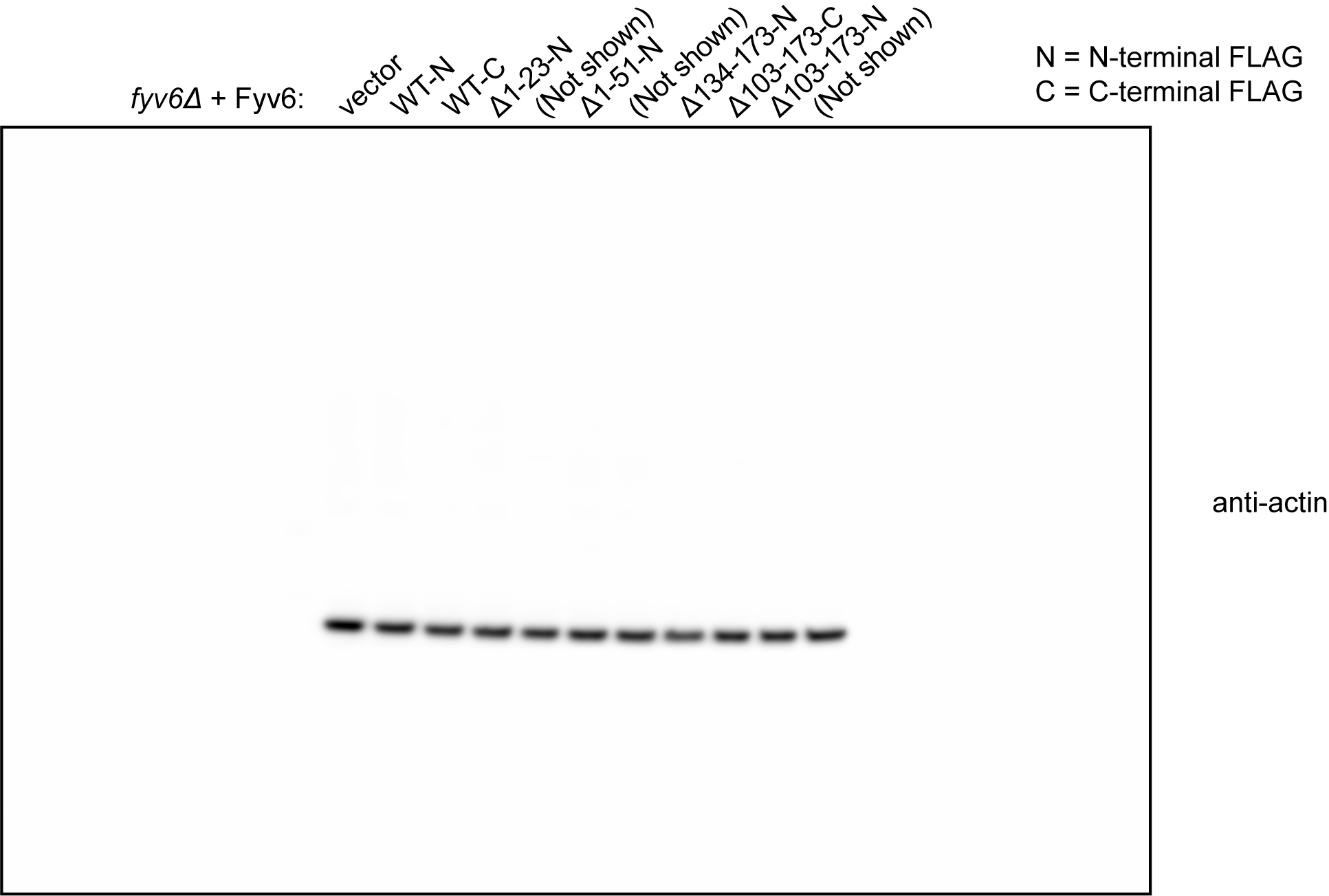

Supplement: Figure 5—figure supplement 1—source data 1. [file elife-100449-fig5-figsupp1-data1.zip › Figure 5-figure supplement 1-source data 1/Figure5-FigureSupplement1-Actin-1-labeled.tif]

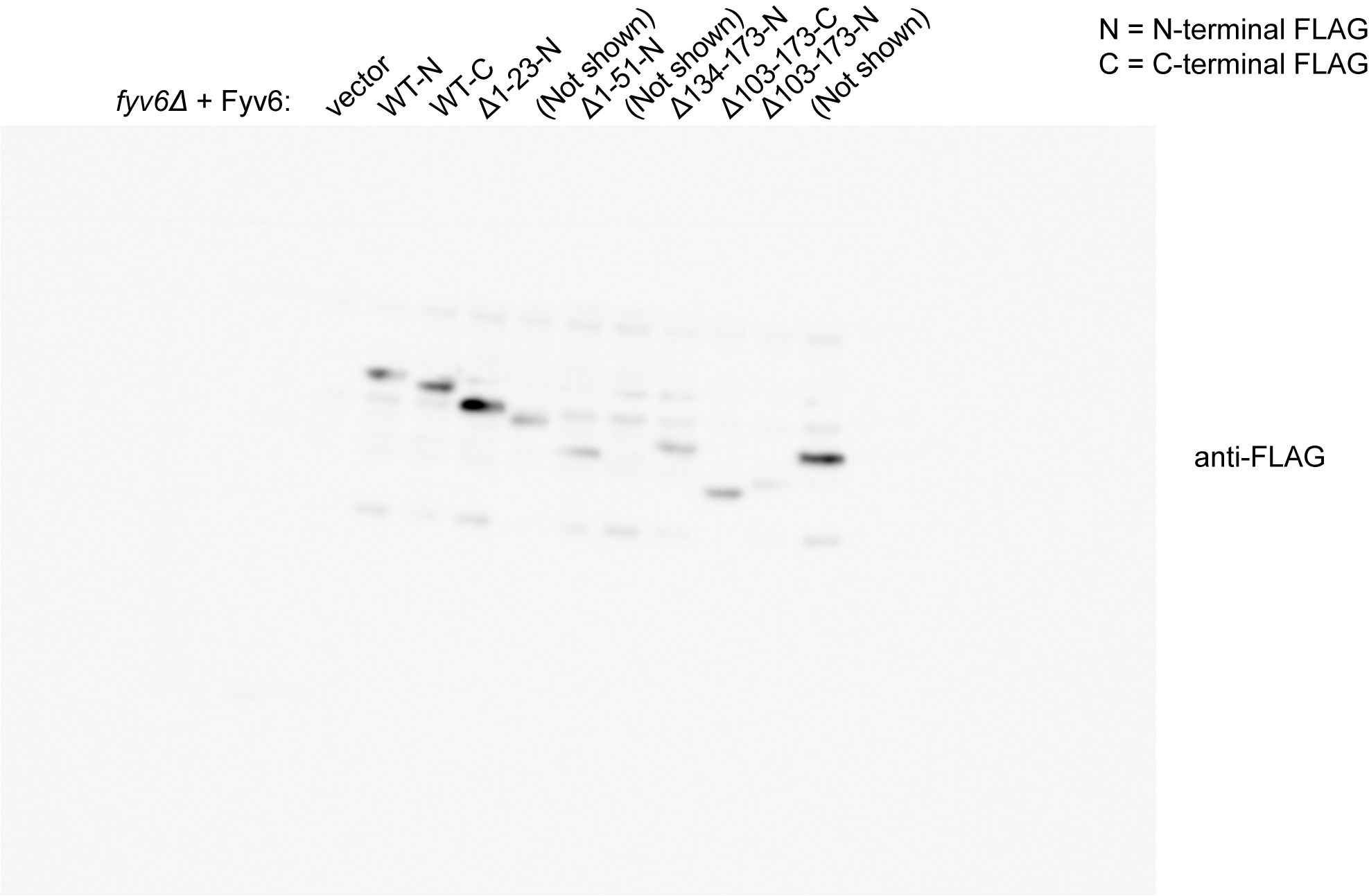

Supplement: Figure 5—figure supplement 1—source data 1. [file elife-100449-fig5-figsupp1-data1.zip › Figure 5-figure supplement 1-source data 1/Figure5-FigureSupplement1-FLAG-1-labeled.tif]

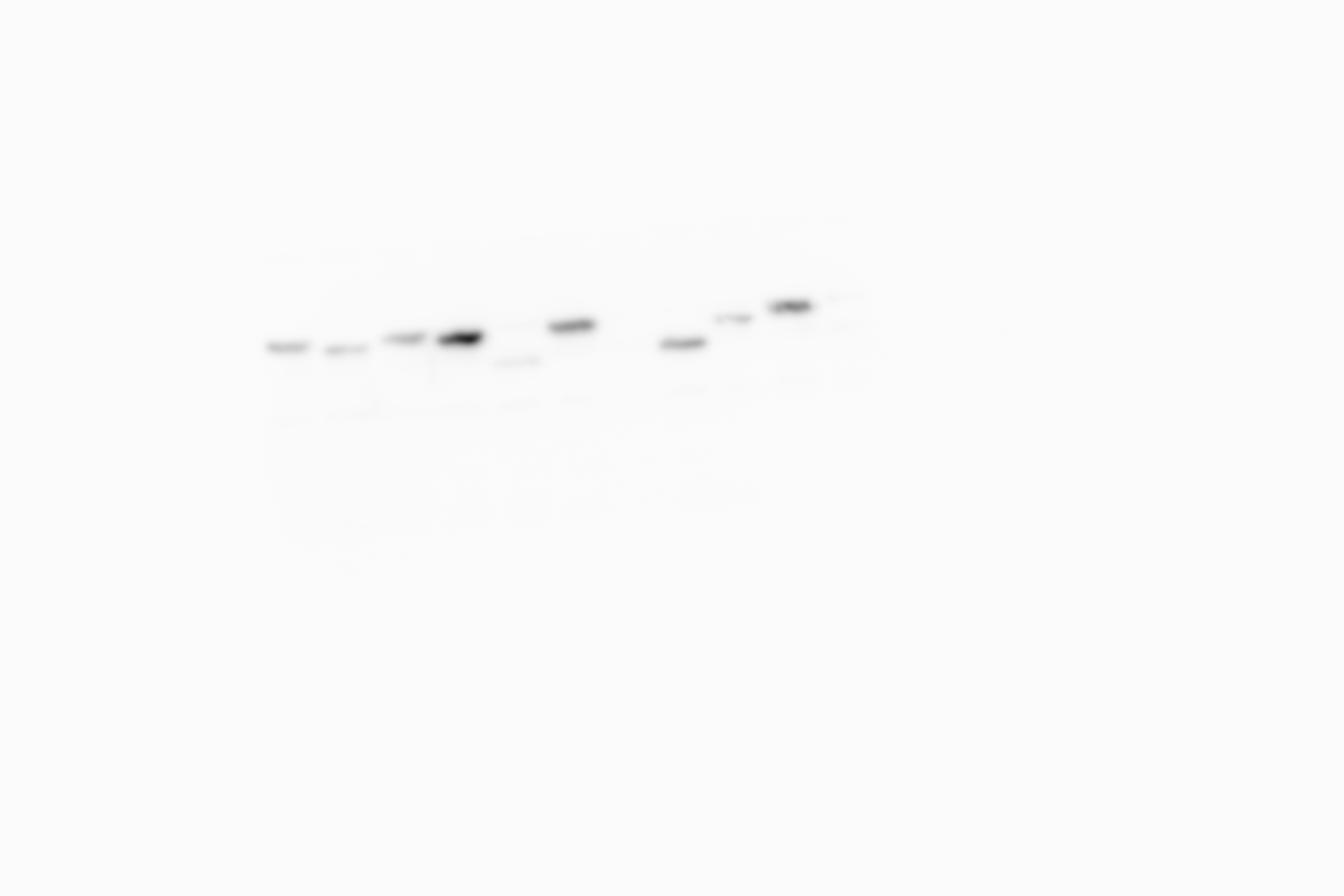

Supplement: Figure 5—figure supplement 1—source data 2. [file elife-100449-fig5-figsupp1-data2.zip › Figure 5-figure supplement 1-source data 2/Figure5-FigureSupplement1-FLAG-2-raw.gel]

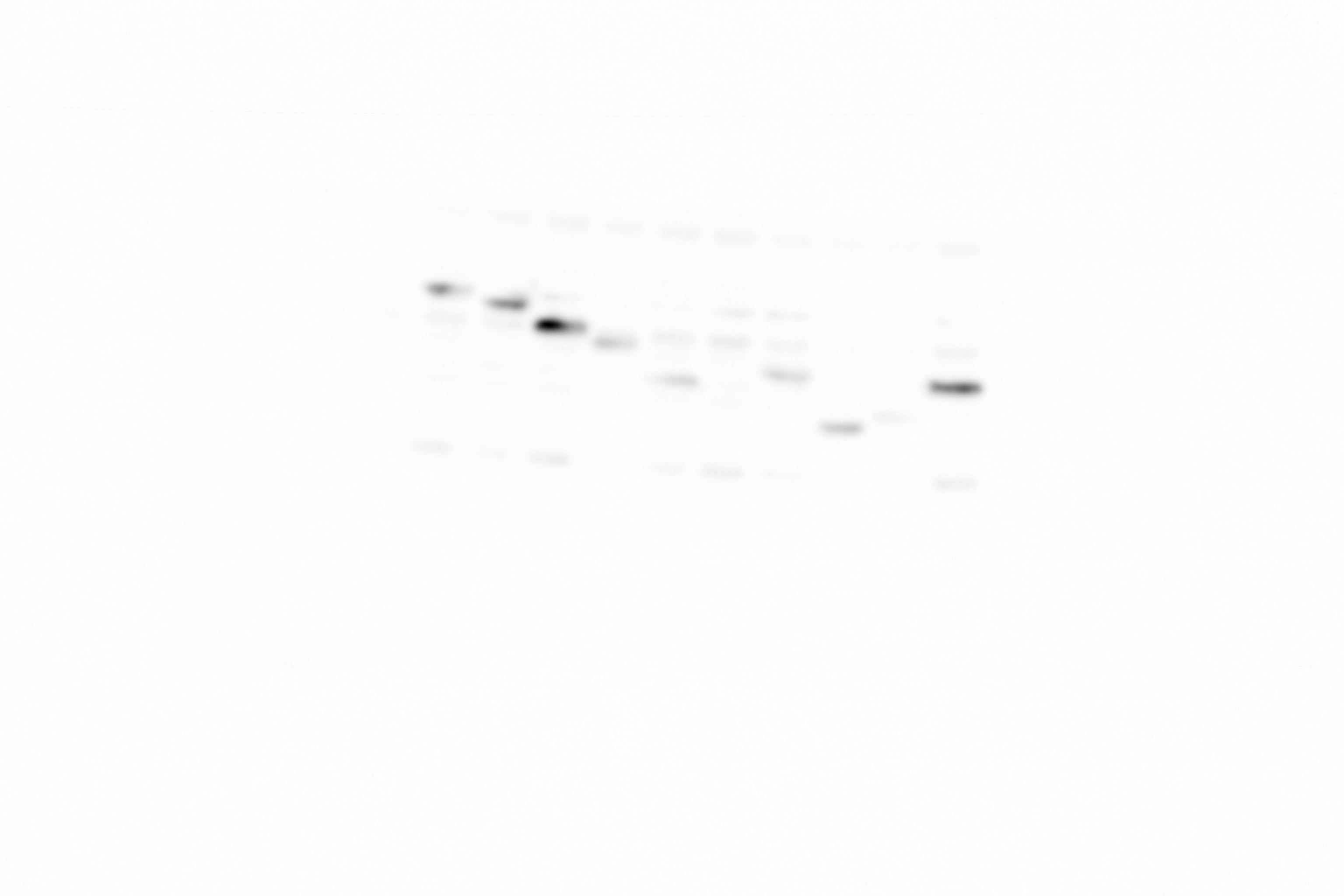

Supplement: Figure 5—figure supplement 1—source data 2. [file elife-100449-fig5-figsupp1-data2.zip › Figure 5-figure supplement 1-source data 2/Figure5-FigureSupplement1-FLAG-1-raw.gel]

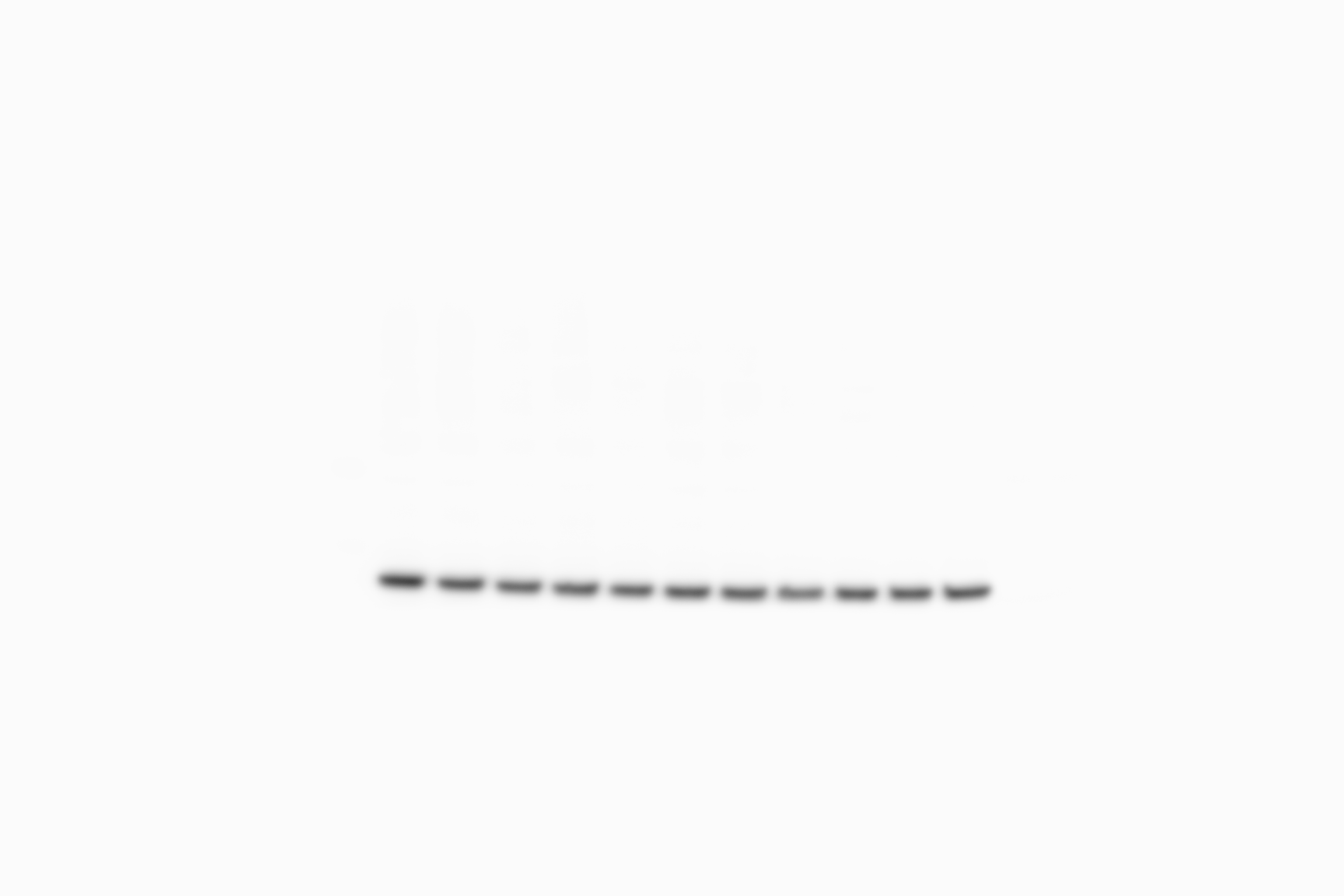

Supplement: Figure 5—figure supplement 1—source data 2. [file elife-100449-fig5-figsupp1-data2.zip › Figure 5-figure supplement 1-source data 2/Figure5-FigureSupplement1-Actin-1-raw.gel]

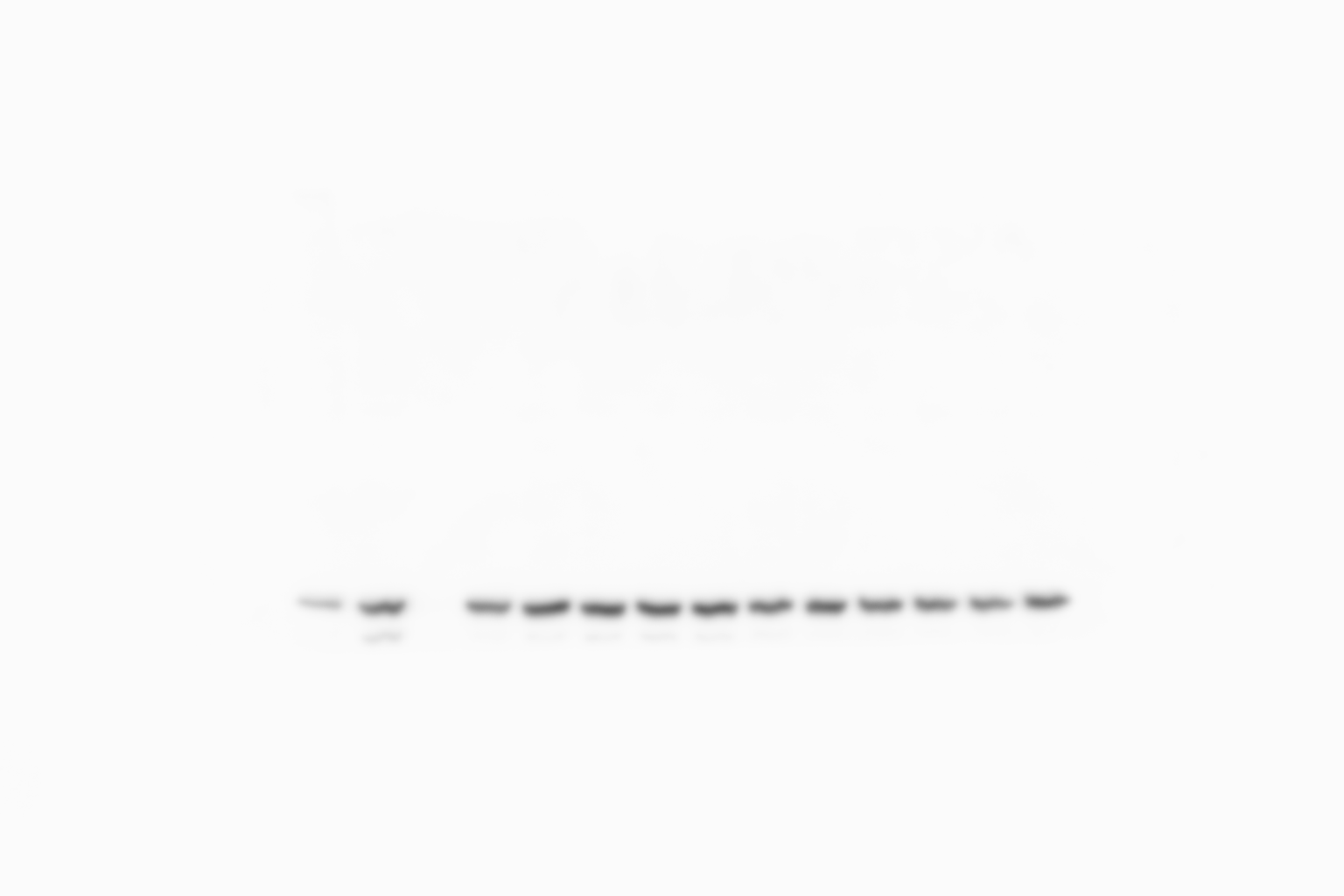

Supplement: Figure 5—figure supplement 1—source data 2. [file elife-100449-fig5-figsupp1-data2.zip › Figure 5-figure supplement 1-source data 2/Figure5-FigureSupplement1-Actin-raw.gel]

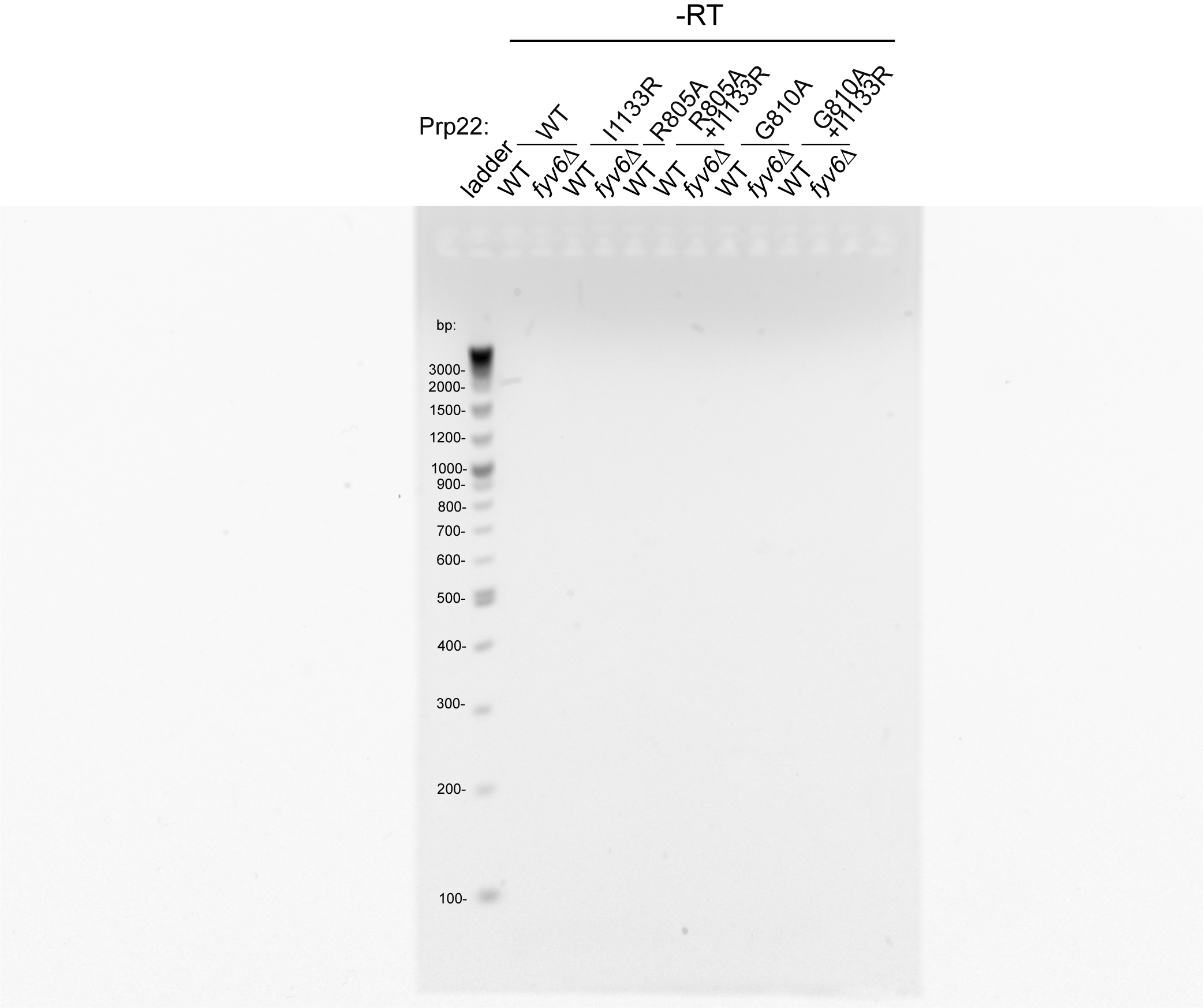

Supplement: Figure 7—source data 1. [file elife-100449-fig7-data1.zip › Figure 7-source data 1/Figure7d-noRT-labeled.tif]

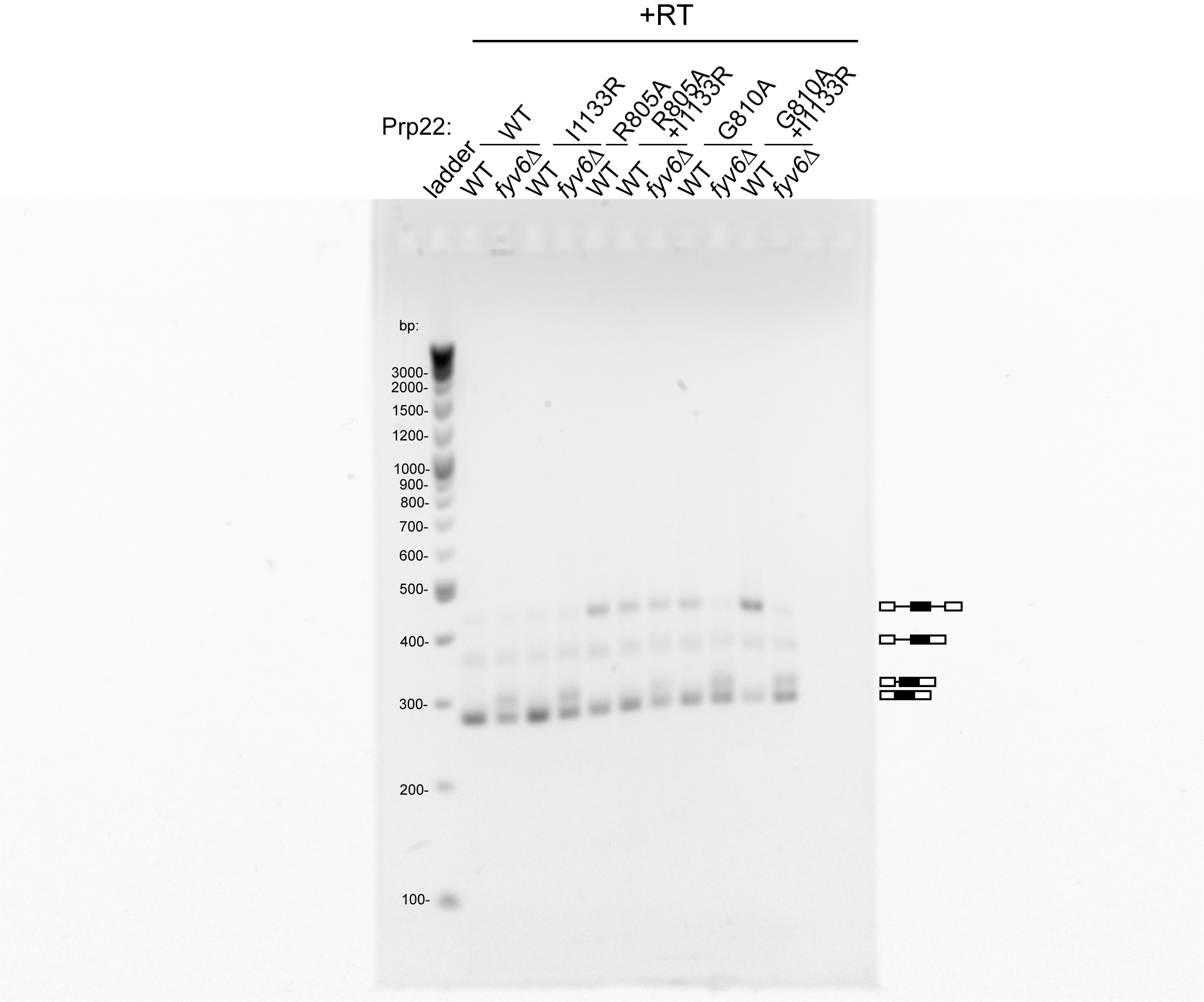

Supplement: Figure 7—source data 1. [file elife-100449-fig7-data1.zip › Figure 7-source data 1/Figure7d-+RT-labeled.tif]

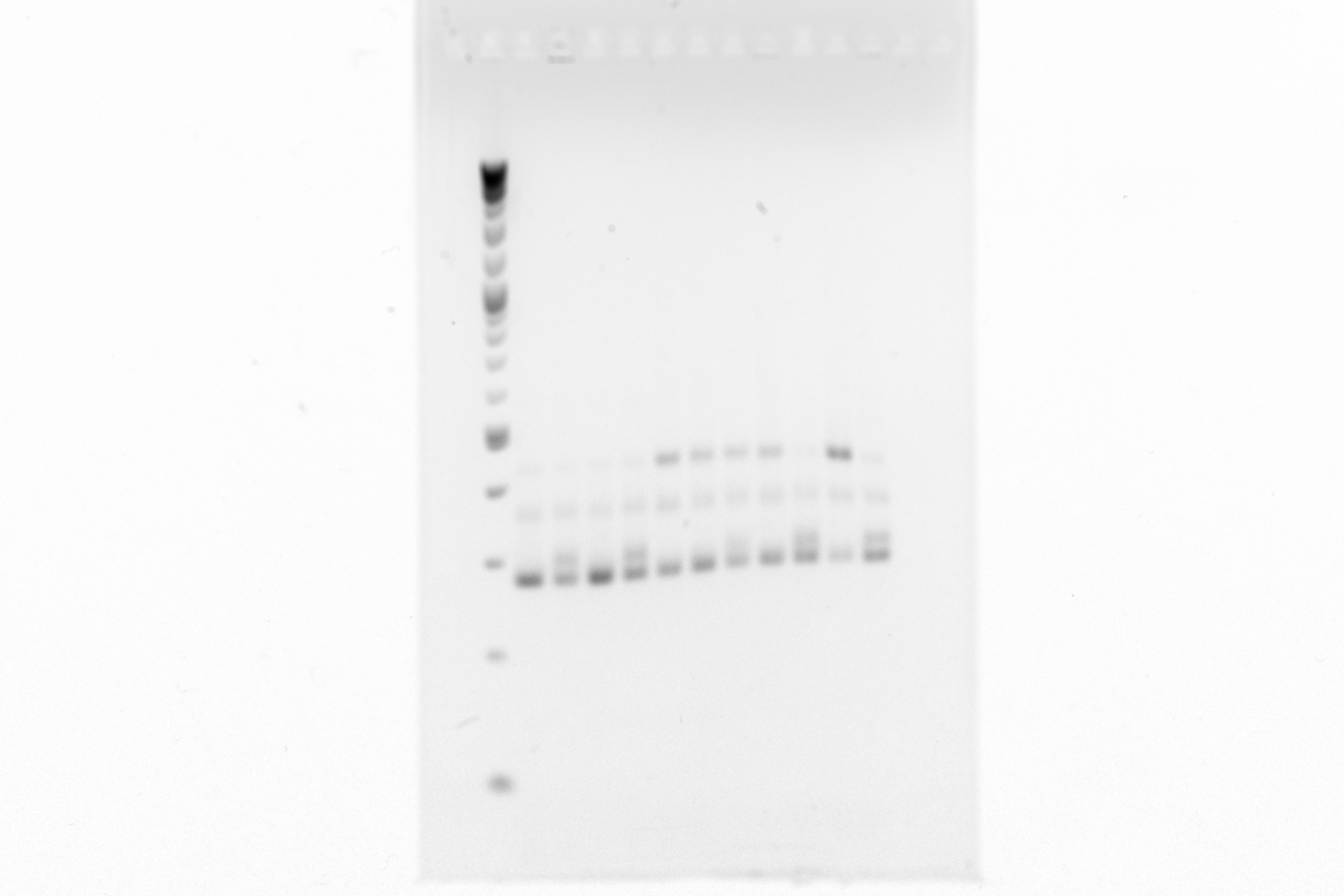

Supplement: Figure 7—source data 2. [file elife-100449-fig7-data2.zip › Figure 7-source data 2/Figure7d-+RT-raw.gel]

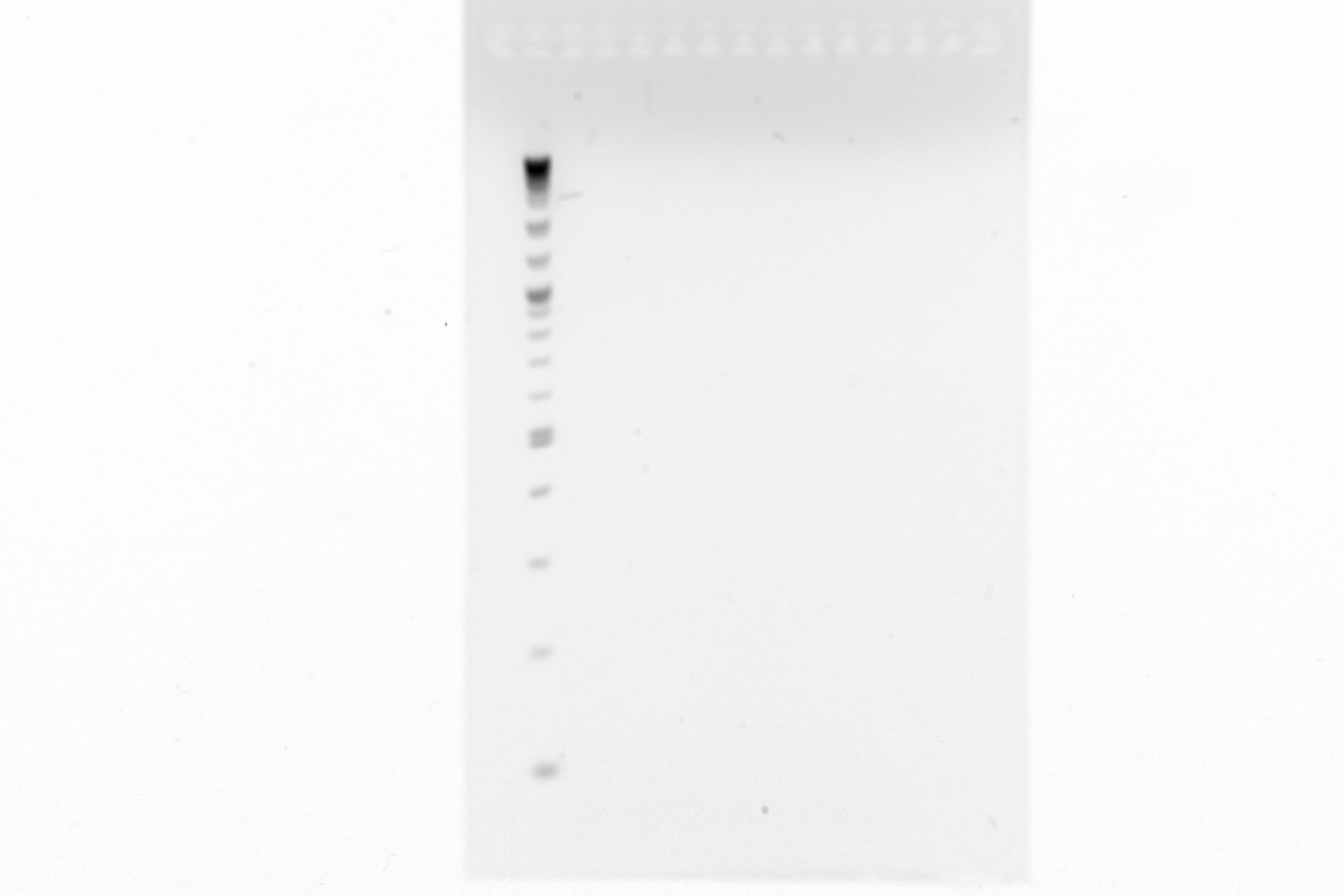

Supplement: Figure 7—source data 2. [file elife-100449-fig7-data2.zip › Figure 7-source data 2/Figure7d-noRT-raw.gel]
