# Supplementary material for: Control of 3′ splice site selection by the yeast splicing factor Fyv6
Source: eLife. 2024 Dec 17;13:RP100449. doi: 10.7554/eLife.100449 (PMC11651659; doi:10.7554/eLife.100449)
Supplement: Supplementary file 2. [file elife-100449-supp2.docx]

**Supplemental File 2. RNA-seq datasets used for analysis.**

| Datasets | Description/Encode # | Figures |
| --- | --- | --- |
| WT-16-12-8, WT-16-1-5, WT-30-12-8, WT-30-1-5, Fyv6-16-12-8, Fyv6-16-1-5, Fyv6-30-12-8, Fyv6-30-1-5, Fyv6-37-12-8, Fyv6-37-1-5 | Temperature shifted RNA-seq datasets for WT *upf1Δ* and *fyv6Δ upf1Δ* yeast strains | Fig. 1 |
| WT-0409, WT-0417, Fyv6-0409, Fyv6-0417 | RNA-seq datasets for WT and fyv6Δ yeast strains | Fig. S1C |
| WT0803, WT0811,  Del0803, Del0811 | RNA-seq datasets for WT upf1Δ and fyv6Δ upf1Δ yeast strains | Fig. S1A,D; Fig. 2 |
