# Supplementary material for: Control of 3′ splice site selection by the yeast splicing factor Fyv6
Source: eLife. 2024 Dec 17;13:RP100449. doi: 10.7554/eLife.100449 (PMC11651659; doi:10.7554/eLife.100449)
Supplement: Supplementary file 3. [file elife-100449-supp3.docx]

**Supplementary File 3. Sequences of introns between the branch point and 3ʹ SS in ACT1-CUP1 reporters.**

| BP-3ʹ SS distance | Intron sequence between branch site and 3ʹ SS* |
| --- | --- |
| 9 nt | **UACUAACA**UCGAUUAUA**UAG** |
| 12 nt | **UACUAACA**UCGAUUUGUUUA**UAG** |
| 15 nt | **UACUAACA**UCGAUUAUAUGUUUA**UAG** |
| 21 nt | **UACUAACA**UCGUUCUUCUUUCCGAUUAUA**UAG** |
| 27 nt | **UACUAACA**UCGUUCUUCUUUCCGAUUAUAUGUUUA**UAG** |
| 38 nt | **UACUAACA**UCGAUUGCUUCAUUCUUUUUGUUGCUAUAUUAUAUGUU**UAG** |
| 42 nt | **UACUAACA**UCGAAACAUUGCUUCAUUCUUUUUGUUGCUAUAUUAUAUGUU**UAG** |
| 46 nt | **UACUAACA**UCGAAACAACAAUUGCUUCAUUCUUUUUGUUGCUAUAUUAUAUGUU**UAG** |
| 50 nt | **UACUAACA**UCGAAACAACAAACGAUUGCUUCAUUCUUUUUGUUGCUAUAUUAUAUGUU**UAG** |

*Branch site and 3ʹ SS sequences in bold
