# Supplementary material for: Control of 3′ splice site selection by the yeast splicing factor Fyv6
Source: eLife. 2024 Dec 17;13:RP100449. doi: 10.7554/eLife.100449 (PMC11651659; doi:10.7554/eLife.100449)
Supplement: Supplementary file 5. [file elife-100449-supp5.docx]

**Supplementary File 5. Mutations identified in *fyv6Δ* suppressor strains selected at 37°C**

| Gene | Chromosome | Position | Substitution | Mutation | Strain(s) | Human Gene | Human Residue |
| --- | --- | --- | --- | --- | --- | --- | --- |
| PRP8 | VIII | 432198 | G->T | S1584Y | 370201 |  | S1512 |
|  |  | 432198 | G->A | S1584F*^ | 372003 |  | S1512 |
|  |  | 431365 | C->G | V1862L | 370801 |  | I1790 |
|  |  | 431347 | C->T | G1868R | 370701 |  | G1796 |
|  |  | 431004 | G->C | T1982S^ | 370501 |  | T1910 |
| SLU7 | IV | 619621 | C->T | E9K | 370301 | SLU7 | N/A |
|  |  | 619576 | C->T | A24R** | 370101 |  | N/A |
|  |  | 619575 | G->C |  |  |  |  |
|  |  | 619570 | C->T | E26K** |  |  | N/A |
|  |  | 619562 | A->C | N28K** |  |  | D58 |
| CDC40 (PRP17) | IV | 1203588 | G->A | P208L | 371102 | CDC40 | A245 |
|  |  | 1203459 | G->A | A251V | 370601 |  | Y367 |
|  |  | 1203266 | C->A | K315N | 371701 |  | K450 |
|  |  | 1203055 | C->A | G386W | 370901 |  | A511 |
| Cef1 | XIII | 693490 | C->T | A37V | 370401 | CDC5L | A35 |
|  |  | 693905 | G->T | M175I | 371201, 371302 |  | Q175 |
|  |  | 693958 | A->C | Q193P | 370804 |  | A193 |
| CLF1 (SYF3) | XII | 384437 | G->A | T33I^^ | 371001 | CRNKL1 | P210 |
| RSE1 | XIII | 175909 | A->T | D799E*^,^ ***^,^**** | 371601, 371901, 372003 | SF3B3 | N/A |
|  |  | 175912 | G->T | D798E*** | 371901 |  | N/A |
|  |  | 175899 | T->C | K803E*** |  |  | N/A |
|  |  | 175896 | C->T | E804K*** |  |  | N/A |
|  |  | 175883 | A->T | I808K*** |  |  | N/A |
| LSR1 | II | 681838 | T->C | A25G | 371802 | RNU2 | A24 |
| PRP22 | V | 182238 | T->G | I1133R** | 371601 | DHX8 | N/A |

*Mutations arose together in strain 372003. **Mutations arose together in strain 370101. ***Mutations arose together in strain 371901. ****Mutations arose together in 371601. ^Second-strongest suppressors of *FYV6* deletion based on colony size. ^^Strongest suppressor of *FYV6* deletion based on colony size.
