# Supplementary material for: Control of 3′ splice site selection by the yeast splicing factor Fyv6
Source: eLife. 2024 Dec 17;13:RP100449. doi: 10.7554/eLife.100449 (PMC11651659; doi:10.7554/eLife.100449)
Supplement: Supplementary file 6. [file elife-100449-supp6.docx]

**Supplementary File 6. Yeast strains used in this study.**

| Strain name | Genotype | Description | Source |
| --- | --- | --- | --- |
| yAAH0434 | MATɑ cup1Δ ura3 his3 trp1 lys2 ade2 leu2 | Cu^2+^ sensitive strain | David Brow |
| yAAH3353 | yAAH0434 + fyv6Δ::hphMX | Cu^2+^ sensitive *fyv6Δ* strain | Lipinski et al., 2023 |
| yAAH3399 | yAAH0434+upf1Δ::KanMX | Cu^2+^ sensitive *upf1Δ*  strain | Lipinski et al., 2023 |
| yAAH3400 | yAAH3353+upf1Δ::KanMX | Cu^2+^ sensitive *fyv6Δ*  *upf1Δ* strain | Lipinski et al., 2023 |
| yAAH3403 | yAAH0434 + dbr1Δ::NatR | Cu^2+^ sensitive *dbr1Δ* strain | This study |
| yAAH3404 | yAAH3353 + dbr1Δ::NatR | Cu^2+^ sensitive *fyv6Δ* *dbr1Δ* strain | This study |
| yAAH3388 | yAAH3353 + pAAH1555 | Cu^2+^ sensitive Fyv6 shuffle strain | This study |
| yAAH3393 | yAAH3353 + pAAH0135 | vector control, contains pRS414 | This study |
| yAAH3419 | yAAH3353 + pAAH1572 | FLAG-Fyv6 (N-terminal tag) | This study |
| yAAH3434 | yAAH3353 + pAAH1577 | FLAG-Fyv6-Δ1-16 | This study |
| yAAH3435 | yAAH3353 + pAAH1578 | FLAG-Fyv6-Δ1-23 | This study |
| yAAH3436 | yAAH3353 + pAAH1579 | FLAG-Fyv6-Δ1-51 | This study |
| yAAH3437 | yAAH3353 + pAAH1580 | FLAG-Fyv6-Δ134-173 | This study |
| yAAH3438 | yAAH3353 + pAAH1581 | FLAG-Fyv6-Δ103-173 | This study |
| yAAH3418 | yAAH3353 + pAAH1573 | Fyv6-FLAG (C-terminal tag) | This study |
| yAAH3442 | yAAH3353 + pAAH1586 | Fyv6-Δ103-173 -FLAG | This study |
| yAAH3455 | yAAH0434 + pAAH1602 | Prp18^WT^ merodiploid | This study |
| yAAH3456 | yAAH0434 + pAAH1603 | Prp18^WT^/Prp18^V191A^ merodiploid | This study |
| yAAH3457 | yAAH0434 + pAAH1604 | Prp18^WT^ /Prp18^S162P^ merodiploid | This study |
| yAAH3458 | yAAH0434 + pAAH1605 | Prp18^WT^ /Prp18^S162P+V191A^ merodiploid | This study |
| yAAH3459 | yAAH3353 + pAAH1602 | Prp18^WT^ merodiploid in *fyv6Δ* strain | This study |
| yAAH3460 | yAAH3353 + pAAH1603 | Prp18^WT^/Prp18^V191A^ merodiploid in *fyv6Δ* strain | This study |
| yAAH3461 | yAAH3353 + pAAH1604 | Prp18^WT^ /Prp18^S162P^ merodiploid in *fyv6Δ* strain | This study |
| yAAH3462 | yAAH3353 + pAAH1605 | Prp18^WT^ /Prp18^S162P+V191A^ merodiploid in *fyv6Δ* strain | This study |
| yAAH3405 | yAAH3403 + pAAH0470 | *dbr1Δ* + WT ACT1-CUP1 (38 nt) | This study |
| yAAH3410 | yAAH3404 + pAAH0470 | *dbr1Δ* *fyv6Δ* + WT ACT1-CUP1 (38 nt) | This study |
| yAAH3498 | yAAH3403 + pAAH1632 | *dbr1Δ* + ACT1-CUP1 (9 nt) | This study |
| yAAH3499 | yAAH3403 + pAAH1633 | *dbr1Δ* + ACT1-CUP1 (12 nt) | This study |
| yAAH3500 | yAAH3403 + pAAH1634 | *dbr1Δ* + ACT1-CUP1 (21 nt) | This study |
| yAAH3501 | yAAH3403 + pAAH1635 | *dbr1Δ* + ACT1-CUP1 (27 nt) | This study |
| yAAH3503 | yAAH3404 + pAAH1633 | *dbr1Δ* *fyv6Δ* + ACT1-CUP1 (9 nt) | This study |
| yAAH3504 | yAAH3404 + pAAH1634 | *dbr1Δ* *fyv6Δ* + ACT1-CUP1 (12 nt) | This study |
| yAAH3505 | yAAH3404 + pAAH1635 | *dbr1Δ* *fyv6Δ* + ACT1-CUP1 (21 nt) | This study |
| yAAH3506 | yAAH3404 + pAAH1632 | *dbr1Δ* *fyv6Δ* + ACT1-CUP1 (27 nt) | This study |
| yAAH3509 | yAAH3403 + pAAH1636 | *dbr1Δ* + ACT1-CUP1 (15 nt) | This study |
| yAAH3510 | yAAH3403 + pAAH1637 | *dbr1Δ* + ACT1-CUP1 (42 nt) | This study |
| yAAH3511 | yAAH3403 + pAAH1638 | *dbr1Δ* + ACT1-CUP1 (46 nt) | This study |
| yAAH3512 | yAAH3403 + pAAH1639 | *dbr1Δ* + ACT1-CUP1 (50 nt) | This study |
| yAAH3513 | yAAH3404 + pAAH1636 | *dbr1Δ* *fyv6Δ* + ACT1-CUP1 (15 nt) | This study |
| yAAH3514 | yAAH3404 + pAAH1637 | *dbr1Δ* *fyv6Δ*+ ACT1-CUP1 (42 nt) | This study |
| yAAH3515 | yAAH3404 + pAAH1638 | *dbr1Δ* *fyv6Δ*+ ACT1-CUP1 (46 nt) | This study |
| yAAH3516 | yAAH3404 + pAAH1639 | *dbr1Δ* *fyv6Δ*+ ACT1-CUP1 (50 nt) | This study |
| yAAH3517 | yAAH0434+syf1Δ::KanMX + pAAH1624 | Cu^2+^ sensitive Syf1 shuffle strain | This study |
| yAAH3518 | yAAH3517+ fyv6Δ::HygR | Cu^2+^ sensitive Syf1 shuffle strain with *fyv6Δ* | This study |
| yAAH3519 | yAAH0434+syf1Δ::KanMX + pAAH1625 | WT Syf1 | This study |
| yAAH3577 | yAAH0434+syf1Δ::KanMX + +pAAH1666 | Syf1 Δ817-859 | This study |
| yAAH3578 | yAAH0434+syf1Δ::KanMX + fyv6Δ::HygR + pAAH1666 | *fyv6Δ* + Syf1 Δ817-859 | This study |
| yAAH3579 | yAAH0434+syf1Δ::KanMX + +pAAH1667 | Syf1 Δ778-859 | This study |
| yAAH3580 | yAAH0434+syf1Δ::KanMX + fyv6Δ::HygR + pAAH1667 | *fyv6Δ* + Syf1 Δ778-859 | This study |
| yAAH3581 | yAAH0434+syf1Δ::KanMX + +pAAH1668 | Syf1 Δ634-859 | This study |
| yAAH3582 | yAAH0434+syf1Δ::KanMX + fyv6Δ::HygR + pAAH1668 | *fyv6Δ* + Syf1 Δ634-859 | This study |
| yAAH3583 | yAAH0434+syf1Δ::KanMX + fyv6Δ::HygR + pAAH1625 (WT Syf1) | *fyv6Δ* + WT Syf1 | This study |
| yAAH3593 | yAAH0434 + cef1Δ::KanMX + pAAH1658 | Cu^2+^ sensitive Cef1 shuffle strain | This study |
| yAAH3594 | yAAH3353 + cef1Δ::KanMX + pAAH1658 | Cu^2+^ sensitive Cef1 shuffle strain with fyv6Δ | This study |
| yAAH3634 | yAAH0434 + cef1Δ::KanMX + pAAH1611 | Cef1^WT^ | This study |
| yAAH3636 | yAAH0434 + cef1Δ::KanMX + pAAH1642 | Cef1^M175I^ | This study |
| yAAH3637 | yAAH0434 + cef1Δ::KanMX + pAAH1612 | Cef1^A37P^ | This study |
| yAAH3638 | yAAH0434 + cef1Δ::KanMX + pAAH1641 | Cef1^A37V^ | This study |
| yAAH3639 | yAAH0434 + cef1Δ::KanMX + pAAH1614 | Cef1^V36R^ | This study |
| yAAH3640 | yAAH0434 + cef1Δ::KanMX + pAAH1613 | Cef1^S48R^ | This study |
| yAAH3641 | yAAH0434 + cef1Δ::KanMX + pAAH1643 | Cef1^Q193P^ | This study |
| yAAH3655 | yAAH3353 + cef1Δ::KanMX + pAAH1612 | *fyv6Δ* Cef1^A37P^ | This study |
| yAAH3656 | yAAH3353 + cef1Δ::KanMX + pAAH1642 | *fyv6Δ* Cef1^M175I^ | This study |
| yAAH3657 | yAAH3353 + cef1Δ::KanMX + pAAH1641 | *fyv6Δ* Cef1^A37V^ | This study |
| yAAH3658 | yAAH3353 + cef1Δ::KanMX + pAAH1614 | *fyv6Δ* Cef1^V36R^ | This study |
| yAAH3659 | yAAH3353 + cef1Δ::KanMX + pAAH1613 | *fyv6Δ* Cef1^S48R^ | This study |
| yAAH3660 | yAAH3353 + cef1Δ::KanMX + pAAH1643 | *fyv6Δ* Cef1^Q193P^ | This study |
| yAAH3661 | yAAH3353 + cef1Δ::KanMX + pAAH1658 | *fyv6Δ* Cef1^WT^ | This study |
| yAAH0117 | ade2, cup1Δ:ura3 his3 leu2 lys2 prp8Δ:lys2 trp1 pJU169:PRP8(URA) | Cu^2+^ sensitive Prp8 shuffle strain | Christine Guthrie |
| yAAH3352 | yAAH117 + fyv6Δ::HygR | Cu^2+^ sensitive *fyv6Δ* Prp8 shuffle strain | Lipinski et al., 2023 |
| yAAH3093 | ade2, cup1Δ:ura3 his3 leu2 lys2 prp8Δ:lys2 trp1 +pAAH1440 | Prp8^WT^ | This study |
| yAAH3683 | ade2, cup1Δ:ura3 his3 leu2 lys2 prp8Δ:lys2 trp1 +pAAH1659 | Prp8^S1584Y^ | This study |
| yAAH3684 | ade2, cup1Δ:ura3 his3 leu2 lys2 prp8Δ:lys2 trp1 +pAAH1660 | Prp8^S1584F^ | This study |
| yAAH3685 | ade2, cup1Δ:ura3 his3 leu2 lys2 prp8Δ:lys2 trp1 +pAAH1661 | Prp8^V1862L^ | This study |
| yAAH3686 | ade2, cup1Δ:ura3 his3 leu2 lys2 prp8Δ:lys2 trp1 +pAAH1662 | Prp8^G1868R^ | This study |
| yAAH3687 | ade2, cup1Δ:ura3 his3 leu2 lys2 prp8Δ:lys2 trp1 +pAAH1663 | Prp8^T1982S^ | This study |
| yAAH3688 | ade2, cup1Δ:ura3 his3 leu2 lys2 prp8Δ:lys2 trp1 fyv6Δ::HygR +pAAH1659 | *fyv6Δ* Prp8^S1584Y^ | This study |
| yAAH3689 | ade2, cup1Δ:ura3 his3 leu2 lys2 prp8Δ:lys2 trp1 fyv6Δ::HygR +pAAH1660 | *fyv6Δ* Prp8^S1584F^ | This study |
| yAAH3690 | ade2, cup1Δ:ura3 his3 leu2 lys2 prp8Δ:lys2 trp1 fyv6Δ::HygR +pAAH1661 | *fyv6Δ* Prp8^V1862L^ | This study |
| yAAH3691 | ade2, cup1Δ:ura3 his3 leu2 lys2 prp8Δ:lys2 trp1 fyv6Δ::HygR +pAAH1662 | *fyv6Δ* Prp8^G1868R^ | This study |
| yAAH3692 | ade2, cup1Δ:ura3 his3 leu2 lys2 prp8Δ:lys2 trp1 fyv6Δ::HygR +pAAH1663 | *fyv6Δ* Prp8^T1982S^ | This study |
| yAAH3693 | ade2, cup1Δ:ura3 his3 leu2 lys2 prp8Δ:lys2 trp1 fyv6Δ::HygR +pAAH1440 | *fyv6Δ* Prp8^WT^ | This study |
| yAAH1930 | MATa ade2 cup1Δ::ura3 his3 leu2 lys2 trp1 ura3 GAL+ prp22Δ::loxP pPrp22 (URA3) | Prp22 Shuffle Strain | Charles Query |
| yAAH1931 | MATa ade2 cup1Δ::ura3 his3 leu2 lys2 trp1 ura3 GAL+ prp22Δ::loxP +pAAH1042 | Prp22^WT^ | Charles Query |
| yAAH3377 | MATa ade2 cup1Δ::ura3 his3 leu2 lys2 trp1 ura3 GAL+ prp22Δ::loxP fyv6Δ::hygR pPrp22 (URA3) | f*yv6Δ* Prp22 Shuffle Strain | Lipinski et al., 2023 |
| yAAH3379 | MATa ade2 cup1Δ::ura3 his3 leu2 lys2 trp1 ura3 GAL+ prp22Δ::loxP fyv6Δ::hygR + pAAH1042 | *fyv6Δ* Prp22^WT^ | Lipinski et al., 2023 |
| yAAH3558 | MATa ade2 cup1Δ::ura3 his3 leu2 lys2 trp1 ura3 GAL+ prp22Δ::loxP +pAAH1648 | Prp22^I1133R^ | This study |
| yAAH3359 | MATa ade2 cup1Δ::ura3 his3 leu2 lys2 trp1 ura3 GAL+ prp22Δ::loxP fyv6Δ::hygR +pAAH1648 | *fyv6Δ* Prp22^I1133R^ | This study |
| yAAH3606 | MATa ade2 cup1Δ::ura3 his3 leu2 lys2 trp1 ura3 GAL+ prp22Δ::loxP +pAAH1665 | Prp22^R805A^ | This study |
| yAAH3607 | MATa ade2 cup1Δ::ura3 his3 leu2 lys2 trp1 ura3 GAL+ prp22Δ::loxP +pAAH1664 | Prp22^G810A^ | This study |
| yAAH3608 | MATa ade2 cup1Δ::ura3 his3 leu2 lys2 trp1 ura3 GAL+ prp22Δ::loxP fyv6Δ::hygR +pAAH1664 | *fyv6Δ* Prp22^G810A^ | This study |
| yAAH3612 | yAAH1930 + pAAH1675 | pre-5-FOA selection Prp22^R805A+I1133R^ | This study |
| yAAH3613 | yAAH1930 + pAAH1674 | pre-5-FOA selection Prp22^G810A+I1133R^ | This study |
| yAAH3614 | yAAH3377 + pAAH1665 | pre-5-FOA selection *fyv6Δ* Prp22^R805A^ | This study |
| yAAH3615 | yAAH3377 + pAAH1675 | pre-5-FOA selection *fyv6Δ* Prp22^R805A+I1133R^ | This study |
| yAAH3616 | yAAH3377 + pAAH1674 | pre-5-FOA selection *fyv6Δ* Prp22^G810A+I1133R^ | This study |
| yAAH3632 | MATa ade2 cup1Δ::ura3 his3 leu2 lys2 trp1 ura3 GAL+ prp22Δ::loxP fyv6Δ::hygR +pAAH1675 | *fyv6Δ* Prp22^R805A+I1133R^ | This study |
| yAAH3633 | MATa ade2 cup1Δ::ura3 his3 leu2 lys2 trp1 ura3 GAL+ prp22Δ::loxP fyv6Δ::hygR +pAAH1674 | *fyv6Δ* Prp22^G810A+I1133R^ | This study |
| yAAH3635 | MATa ade2 cup1Δ::ura3 his3 leu2 lys2 trp1 ura3 GAL+ prp22Δ::loxP +pAAH1674 | Prp22^G810A+I1133R^ | This study |
| yAAH3662 | MATa ade2 cup1Δ::ura3 his3 leu2 lys2 trp1 ura3 GAL+ prp22Δ::loxP +pAAH1675 | Prp22^R805A+I1133R^ | This study |
| yAAH3663 | yAAH1931 + pAAH0470 | Prp22^WT^ + WT ACT1-CUP1 | This study |
| yAAH3664 | yAAH1931 + pAAH0526 | Prp22^WT^ + ACT1-CUP1 U301G | This study |
| yAAH3665 | yAAH1931 + pAAH0527 | Prp22^WT^ + ACT1-CUP1 A302U | This study |
| yAAH3666 | yAAH3607 + pAAH0470 | Prp22^G810A^ + WT ACT1-CUP1 | This study |
| yAAH3667 | yAAH3607 + pAAH0527 | Prp22^G810A^ + ACT1-CUP1 A302U | This study |
| yAAH3694 | yAAH3607 + pAAH0526 | Prp22^G810A^ + ACT1-CUP1 U301G | This study |
| yAAH3695 | yAAH3379 + pAAH0470 | *fyv6Δ* Prp22^WT^ + WT ACT1-CUP1 | This study |
| yAAH3696 | yAAH3379 + pAAH0526 | *fyv6Δ* Prp22^WT^ + ACT1-CUP1 U301G (gAG) | This study |
| yAAH3697 | yAAH3379 + pAAH0527 | *fyv6Δ* Prp22^WT^ + ACT1-CUP1 A302U (UuG) | This study |
| yAAH3698 | yAAH3608 + pAAH0470 | *fyv6Δ* Prp22^WT^ + WT ACT1-CUP1 | This study |
| yAAH3699 | yAAH3608 + pAAH0526 | *fyv6Δ* Prp22^WT^ + ACT1-CUP1 U301G (gAG) | This study |
| yAAH3700 | yAAH3608 + pAAH0527 | *fyv6Δ* Prp22^WT^ + ACT1-CUP1 A302U (UuG) | This study |
| yAAH3701 | yAAH1931 + pAAH0880 | Prp22^WT^ + ACT1-CUP1 BSG | This study |
| yAAH3702 | yAAH3607 + pAAH0880 | Prp22^G810A^ + ACT1-CUP1 BSG | This study |
| yAAH3703 | yAAH3379 + pAAH0880 | *fyv6Δ* Prp22^WT^ + ACT1-CUP1 BSG | This study |
| yAAH3704 | yAAH3608 + pAAH0880 | *fyv6Δ* Prp22^G810A^ + ACT1-CUP1 BSG | This study |
| yAAH3748 | yAAH3559 + pAAH0470 | *fyv6Δ* Prp22^I1133R^  + WT ACT1-CUP1 | This study |
| yAAH3749 | yAAH3559 + pAAH0526 | *fyv6Δ* Prp22^I1133R^  + ACT1-CUP1 U301G (gAG) | This study |
| yAAH3750 | yAAH3559 + pAAH0527 | *fyv6Δ* Prp22^I1133R^  + ACT1-CUP1 A302U (UuG) | This study |
| yAAH3751 | yAAH3559 + pAAH0880 | *fyv6Δ* Prp22^I1133R^  + ACT1-CUP1 BSG | This study |
| yAAH3771 | yAAH3635 + pAAH0470 | Prp22^G810A+I1133R^ + WT ACT1-CUP1 | This study |
| yAAH3772 | yAAH3635 + pAAH0526 | Prp22^G810A+I1133R^ + ACT1-CUP1 U301G | This study |
| yAAH3773 | yAAH3635 + pAAH0527 | Prp22^G810A+I1133R^ + ACT1-CUP1 A302U | This study |
| yAAH3774 | yAAH3635 + pAAH0880 | Prp22^G810A+I1133R^ + ACT1-CUP1 BSG | This study |
| yAAH3779 | yAAH3558 + pAAH0470 | Prp22^I1133R^  + WT ACT1-CUP1 | This study |
| yAAH3780 | yAAH3558 + pAAH0526 | Prp22^I1133R^  + ACT1-CUP1 U301G | This study |
| yAAH3781 | yAAH3558 + pAAH0527 | Prp22^I1133R^  + ACT1-CUP1 A302U | This study |
| yAAH3782 | yAAH3558 + pAAH0880 | Prp22^I1133R^  + ACT1-CUP1 BSG | This study |
| yAAH3783 | yAAH3633 + pAAH0470 | *fyv6Δ* Prp22^G810A+I1133R^ + WT ACT1-CUP1 | This study |
| yAAH3784 | yAAH3633 + pAAH0526 | *fyv6Δ* Prp22^G810A+I1133R^ + ACT1-CUP1 U301G | This study |
| yAAH3785 | yAAH3633 + pAAH0527 | *fyv6Δ* Prp22^G810A+I1133R^ + ACT1-CUP1 A302U | This study |
| yAAH3786 | yAAH3633 + pAAH0880 | *fyv6Δ* Prp22^G810A+I1133R^ + ACT1-CUP1 BSG | This study |
| BCY123 | MATa pep4::HIS3 prb1::LEU2 bar1::HIS6 lys2::GAL1/10-GAL4 can1 ade2 trp1 ura3 his3 leu2-3,112 | Protease-deficient yeast protein expression strain | Galej et al., 2013 |
