# Supplementary material for: Control of 3′ splice site selection by the yeast splicing factor Fyv6
Source: eLife. 2024 Dec 17;13:RP100449. doi: 10.7554/eLife.100449 (PMC11651659; doi:10.7554/eLife.100449)
Supplement: Supplementary file 7. [file elife-100449-supp7.docx]

**Supplementary File 7**. **Plasmids used in this study.**

| **Plasmid ID** | **Plasmid name** | **Description** | **Source** |
| --- | --- | --- | --- |
| pAAH0135 | pRS414 | CEN6/ARSH4 TRP1  Vector for Fyv6 plasmids | Mumberg et al., 1995  (ATCC# 87519) |
| pAAH1555 | pRS416-Fyv6 | FYV6 +/- ~250 bp (URA3 CEN6/ARSH4), used for Fyv6 shuffle strain | This study |
| pAAH1556 | pRS414-Fyv6 | FYV6 +/- ~250 bp (TRP1 CEN6/ARSH4) | This study |
| pAAH1572 | pRS414-FLAG-Fyv6 | N-terminally FLAG tagged FYV6 +/- ~250 bp (TRP1 CEN6/ARSH4) | This study |
| pAAH1577 | pRS414-FLAG-Fyv6-Δ1-16 | FLAG-Fyv6 Δ1-16 (TRP1 CEN ARS); in Fyv6 Δ1-16 truncation strain | This study |
| pAAH1578 | pRS414-FLAG-Fyv6-Δ1-23 | FLAG-Fyv6 Δ1-23 (TRP1 CEN ARS); in Fyv6 Δ1-23 truncation strain | This study |
| pAAH1579 | pRS414-FLAG-Fyv6-Δ1-51 | FLAG-Fyv6 Δ1-51 (TRP1 CEN ARS); in Fyv6 Δ1-51 truncation strain | This study |
| pAAH1580 | pRS414-FLAG-Fyv6-Δ134-173 | FLAG-Fyv6 Δ134-173 (TRP1 CEN ARS); in Fyv6 Δ134-173 truncation strain | This study |
| pAAH1581 | pRS414-FLAG-Fyv6-Δ103-173 | FLAG-Fyv6 Δ103-173 (TRP1 CEN ARS); in Fyv6 Δ103-173 truncation strain | This study |
| pAAH1573 | pRS414-Fyv6-FLAG | C-terminally FLAG tagged FYV6 +/- ~250 bp (TRP1 CEN6/ARSH4) | This study |
| pAAH1586 | pRS414-Fyv6-Δ103-173 -FLAG | Fyv6 Δ103-173-FLAG (TRP1 CEN ARS); in Fyv6 Δ103-173 truncation C-terminally FLAG tagged strain | This study |
| pAAH1602 | p360-Prp18 WT | Prp18^WT^ (URA3 CEN) | Aronova et al., 2007; gift from Beate Schwer |
| pAAH1603 | p360-Prp18-11 | Prp18^V191A^ (URA3 CEN) | Aronova et al., 2007; gift from Beate Schwer |
| pAAH1604 | p360-Prp18-18 | Prp18^S162P^ (URA3 CEN) | Aronova et al., 2007; gift from Beate Schwer |
| pAAH1605 | p360-Prp18-11/18 | Prp18^S162P+V191A^ (URA3 CEN) | Aronova et al., 2007; gift from Beate Schwer |
| pAAH0470 | ACT1-CUP1 WT | WT ACT1-CUP1 reporter; 38 nt BP-3ʹ SS spacing. (GAP promoter, LEU2) | Gift from Charles Query. |
| pAAH1632 | ACT1-CUP1-9nt | ACT1-CUP1 reporter with 9 nt BP-3ʹ SS spacing. (GAP promoter, LEU2) | This study |
| pAAH1633 | ACT1-CUP1-12nt | ACT1-CUP1 reporter with 12 nt BP-3ʹ SS spacing. (GAP promoter, LEU2) | This study |
| pAAH1634 | ACT1-CUP1-21nt | ACT1-CUP1 reporter with 21 nt BP-3ʹ SS spacing. (GAP promoter, LEU2) | This study |
| pAAH1635 | ACT1-CUP1-27nt | ACT1-CUP1 reporter with 27 nt BP-3ʹ SS spacing. (GAP promoter, LEU2) | This study |
| pAAH1636 | ACT1-CUP1-15nt | ACT1-CUP1 reporter with 27 nt BP-3ʹ SS spacing. (GAP promoter, LEU2) | This study |
| pAAH1637 | ACT1-CUP1-42nt | ACT1-CUP1 reporter with 42 nt BP-3ʹ SS spacing. (GAP promoter, LEU2) | This study |
| pAAH1638 | ACT1-CUP1-46nt | ACT1-CUP1 reporter with 46 nt BP-3ʹ SS spacing. (GAP promoter, LEU2) | This study |
| pAAH1639 | ACT1-CUP1-50nt | ACT1-CUP1 reporter with 50 nt BP-3ʹ SS spacing. (GAP promoter, LEU2) | This study |
| pAAH1624 | pRS416-Syf1 | SYF1 +/- ~275 bp (URA3 CEN6/ARSH4), used for SYF1 shuffle strain | This study |
| pAAH1625 | pRS414-Syf1 | SYF1 +/- ~275 bp (TRP1 CEN6/ARSH4) | This study |
| pAAH1666 | pRS414-Syf1Δ817-859 | SYF1 Δ817-859 (TRP1 CEN6/ARSH4) | This study |
| pAAH1667 | pRS414-Syf1Δ778-859 | SYF1 Δ778-859 (TRP1 CEN6/ARSH4) | This study |
| pAAH1668 | pRS414-Syf1Δ634-859 | SYF1 Δ634-859 (TRP1 CEN6/ARSH4) | This study |
| pAAH1611 | pRS314-Cef1-WT | Cef1^WT^ (TRP1 CEN) | Query and Konarska, 2012 ; Gift from Charles Query. |
| pAAH1612 | pRS314-Cef1-A37P | Cef1^A37P^ (TRP1 CEN) | Query and Konarska, 2012; Gift from Charles Query. |
| pAAH1613 | pRS314-Cef1-S48R | Cef1^S48R^ (TRP1 CEN) | Query and Konarska, 2012; Gift from Charles Query. |
| pAAH1614 | pRS314-Cef1-9-8 | Cef1^V36R^ (TRP1 CEN) | Query and Konarska, 2012; Gift from Charles Query. |
| pAAH1641 | pRS314-Cef1-A37V | Cef1^A37V^ (TRP1 CEN) | This study |
| pAAH1642 | pRS314-Cef1-M175I | Cef1^M175I^ (TRP1 CEN) | This study |
| pAAH1643 | pRS314-Cef1-Q193P | Cef1^Q193P^ (TRP1 CEN) | This study |
| pAAH1658 | PRS316-Cef1 | WT Cef1 (URA3 CEN) | Query and Konarska, 2012; Gift from Charles Query. |
| pAAH1440 | pRS424-Prp8 | WT Prp8 (full length) (TRP1 2μ) | This study |
| pAAH1659 | pRS424-Prp8-S1584Y | Prp8 S1584Y (TRP1 2μ) | This study |
| pAAH1660 | pRS424-Prp8-S1584F | Prp8 S1584F (TRP1 2μ) | This study |
| pAAH1661 | pRS424-Prp8-V1862L | Prp8 V1862L (TRP1 2μ) | This study |
| pAAH1662 | pRS424-Prp8-G1868R | Prp8 G1868R (TRP1 2μ) | This study |
| pAAH1663 | pRS424-Prp8-T1982S | Prp8 T1982S (TRP1 2μ) | This study |
| pAAH1042 | pPrp22-WT | WT Prp22 (TRP1 CEN) | Gift from Charles Query. |
| pAAH1648 | pPrp22-I1133R | Prp22 I1133R (TRP1 CEN) | This study |
| pAAH1664 | pPrp22-G810A | Prp22 G810A (TRP1 CEN) | Schwer and Meszaros, 2000; Gift from Beate Schwer |
| pAAH1665 | pPrp22-R805A | Prp22 R805A (TRP1 CEN) | Schwer and Meszaros, 2000; Gift from Beate Schwer |
| pAAH1674 | pPrp22- G810A+I1133R | Prp22 G810A+I1133R (TRP1 CEN) | This study |
| pAAH1675 | pPrp22-R805A+I1133R | Prp22 R805A+I1133R (TRP1 CEN) | This study |
| pRS424-CBP-His-TEV-Prp22-S635A | pRS424-CBP-His-TEV-Prp22-S635A | GAL/GAPDH promoter, Prp22 expression (TRP1 2µ) | This study |
| pRS426-CBP-His-TEV-Prp22-S635A | pRS426-CBP-His-TEV-Prp22-S635A | GAL/GAPDH promoter, Prp22 expression (URA3 2µ) | This study |
| pAAH0880 | ACT1-CUP1 BSG (A259G) | BS A259G reporter used for ACT1-CUP1 assays. (GAP promoter, LEU2) | Gift from Charles Query. |
| pAAH0526 | ACT1-CUP1 U301G | 3ʹ SS gAG reporter used for ACT1-CUP1 assays. (GAP promoter, LEU2) | Gift from Charles Query. |
| pAAH0527 | ACT1-CUP1 A302U | 3ʹ SS UuG reporter used for ACT1-CUP1 assays. (GAP promoter, LEU2) | Gift from Charles Query. |
