# Supplementary material for: Control of 3′ splice site selection by the yeast splicing factor Fyv6
Source: eLife. 2024 Dec 17;13:RP100449. doi: 10.7554/eLife.100449 (PMC11651659; doi:10.7554/eLife.100449)
Supplement: Supplementary file 8. [file elife-100449-supp8.docx]

**Supplementary File 8. Oligonucleotides used in this study.**

| **Designation** | **Sequence (5′ to 3′)** | **Use** | **Source or reference** |
| --- | --- | --- | --- |
| SUS1-exon1 | TGGATACTGCGCAATTAAAGAGTC | RT-PCR primer | Hossain et al., 2009; doi: 10.1261/rna.1540409 |
| SUS1-exon3 | TCATTGTGTATCTACAATCTCTTCAAG | RT-PCR primer | Hossain et al., 2009; doi: 10.1261/rna.1540409 |
| YOS1-F | AGT ACT GAG CGA AGA AAG | RT-PCR primer | This paper |
| YOS1-R | CAT CCC AAT AGT AAT TCA TAA AC | RT-PCR primer | This paper |
| RPS18A_fwd | ACAAGGTTCCTTCCAACACA | RT-PCR primer | This paper |
| RPS18A_rev | TAACGACGACCAACACCCTT | RT-PCR primer | This paper |
| CGI121_fwd | GACAAAGAGCAATTGAGGACGA | RT-PCR primer | This paper |
| CGI121_rev | CGTCTTGGGGCTTGAAATCA | RT-PCR primer | This paper |
| NMD2_fwd | CTGCATTGATAATACATTGGACAGA | RT-PCR primer | This paper |
| NMD2_rev | GAACCTTTCACAAAACCCTTCT | RT-PCR primer | This paper |
| OST5_fwd | CAAGGTTTACAATGACTTATGAACA | RT-PCR primer | This paper |
| OST5_rev | ACAGGGCAGATGATAATACAGC | RT-PCR primer | This paper |
| DID4_fwd | GGAAAGAATGTCACTCCGCA | RT-PCR primer | This paper |
| DID4_rev | CTTGCCCATTCTTTGCCGAT | RT-PCR primer | This paper |
| RPS7B_fwd | TCGCCAAGACCTTCATCGAT | RT-PCR primer | This paper |
| RPS7B_rev | AGACAAAGCTGGAACTGGGA | RT-PCR primer | This paper |
| yU6 | /5IRD700/GAACTGCTGATCATCTCTG | For primer extension | Xu and Query, 2007; doi: 10.1016/j.molcel.2007.09.022 |
| YAC6 | /5IRD700/GGCACTCATGACCTTC | For primer extension | Siatecka et al., 1999; doi: 10.1101/gad.13.15.1983 |
| UBC4-complementary oligo | ATGAAGTAGGTGGAT | for RNase H cleavage | Wilkinson et al., 2017; DOI: 10.1126/science.aar3729 |
